# Harnessing 12-lead ECG and MRI data to personalise repolarisation profiles in cardiac digital twin models for enhanced virtual drug testing

**DOI:** 10.1016/j.media.2024.103361

**Published:** 2024-10-18

**Authors:** Julia Camps, Zhinuo Jenny Wang, Ruben Doste, Lucas Arantes Berg, Maxx Holmes, Brodie Lawson, Jakub Tomek, Kevin Burrage, Alfonso Bueno-Orovio, Blanca Rodriguez

**Affiliations:** 1 University of Oxford, Oxford, United Kingdom; 2 Queensland University of Technology, Brisbane, Australia

**Keywords:** Cardiac digital twin, Precision cardiology, Virtual therapy evaluation, reaction-Eikonal model, Monodomain model, Cardiac Magnetic Resonance, Electrocardiogram, Uncertainty quantification, Open-source

## Abstract

Cardiac digital twins are computational tools capturing key functional and anatomical characteristics of patient hearts for investigating disease phenotypes and predicting responses to therapy. When paired with large-scale computational resources and large clinical datasets, digital twin technology can enable virtual clinical trials on virtual cohorts to fast-track therapy development. Here, we present an open-source automated pipeline for personalising ventricular electrophysiological function based on routinely acquired magnetic resonance imaging (MRI) data and the standard 12-lead electrocardiogram (ECG).

Using MRI-based anatomical models, a sequential Monte-Carlo approximate Bayesian computational inference method is extended to infer electrical activation and repolarisation characteristics from the ECG. Fast simulations are conducted with a reaction-Eikonal model, including the Purkinje network and biophysically-detailed subcellular ionic current dynamics for repolarisation. For each patient, parameter uncertainty is represented by inferring an envelope of plausible ventricular models rather than a single one, which means that parameter uncertainty can be propagated to therapy evaluation. Furthermore, we have developed techniques for translating from reaction-Eikonal to monodomain simulations, which allows more realistic simulations of cardiac electrophysiology. The pipeline is demonstrated in three healthy subjects, where our inferred pseudo-diffusion reaction-Eikonal models reproduced the patient’s ECG with a median Pearson’s correlation coefficient of 0.9, and then translated to monodomain simulations with a median correlation coefficient of 0.84 across all subjects. We then demonstrate our digital twins for virtual evaluation of Dofetilide with uncertainty quantification. These evaluations using our cardiac digital twins reproduced dose-dependent QTc and T peak to T end prolongations that are in keeping with large population drug response data.

The methodologies for cardiac digital twinning presented here are a step towards personalised virtual therapy testing and can be scaled to generate virtual populations for clinical trials to fast-track therapy evaluation. The tools developed for this paper are open-source, documented, and made publicly available.

## Graphical abstract

**Graphical abstract:**
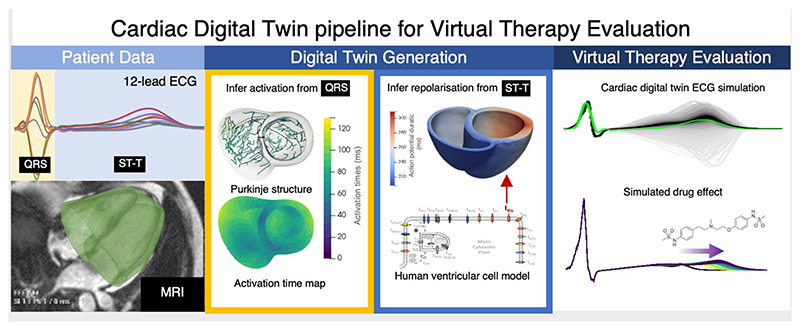

## 1 Introduction

Significant differences in cardiac anatomy and electrophysiological function in the human population drive the need for a precision medicine approach when developing cardiac therapies (Antman & Loscalzo, 2016). The cardiac digital twin is an emerging paradigm describing a suite of tools that continuously and coherently integrate patient data to produce virtual hearts that evolve with their ‘twin’ to help realise the vision of precision medicine in cardiology (Corral-Acero et al., 2020; Niederer et al., 2021). For such cardiac digital twin technologies to be useful for therapy development, computational modelling choices should ensure that the key therapy targets are mathematically represented in sufficient detail so that virtual predictions of therapy outcomes are mechanistic, relevant, and trustworthy (Arevalo et al., 2016; Tomek et al., 2019; Doste et al., 2020). Additionally, the digital twin should not be a single personalised model, but a ‘physiological envelop’ of models that takes into account uncertainties from the input clinical data, the experimentally derived model parameters, and simulator uncertainties when predicting therapeutic outcomes (Mirams et al., 2020). With the advent of exascale computing (Hoekstra et al., 2019) and Big Data in healthcare (Littlejohns et al., 2020), cardiac digital twin technology should be easily scalable to take advantage of the wealth of available data to enable virtual cohorts for *in silico* clinical trials (Passini et al., 2017; Musuamba et al., 2021; Dasi et al., 2022; Roney et al., 2022; Fassina et al., 2023). The availability of open-source cardiac digital twin solutions would fast-track translation by democratising access and enabling community efforts for verification and validation. Existing open-source solutions for generating cardiac digital twins are limited to personalisation of activation properties only (Camps et al., 2024), while no open-source solutions are yet available for personalisation of repolarisation and virtual therapy and disease evaluation. Therefore, in this study we aim to develop a fast and scalable open-source cardiac digital twin generation pipeline to produce a physiological envelope of biophysically detailed models of each subject’s heart, which propagates model and data uncertainties to therapeutic predictions, using routinely-acquired magnetic resonance imaging (MRI) and 12-lead electrocardiograms (ECG).

The ECG encodes information on depolarisation and repolarisation properties relevant to investigations into disease mechanisms as well as drug safety and efficacy evaluations: the QRS complex of the ECG reflects the activation pattern, while the ST segment and the T wave of the ECG comprise information on spatial heterogeneities in repolarisation and action potential duration (APD) (Szentadrassy et al., 2005; Opthof et al., 2017). Repolarisation heterogeneities are underpinned by a complex interplay of subcellular ionic current dynamics (Szentadrassy et al., 2005; Opthof et al., 2017), which are altered by drugs, such as Dofetilide. Therefore, for drug and disease investigations, retaining representation of ionic currents as in the human biophysically-detailed cellular ventricular model by Tomek et al., (2019) is crucial in the context of drug, and disease and potential target investigations.

In this paper, we present an open-source cardiac digital twin generation pipeline to infer ventricular electrophysiological properties from the 12-lead ECG with MRI-derived anatomy, using the human biophysically-detailed cellular ventricular model by Tomek et al., (2019), to ensure suitability for drug and disease evaluation with uncertainty quantification. The inference process requires tens of thousands of ventricular simulations per subject in a reasonable timeframe, building on works by Camps et al., (2021, 2024). Therefore, reducing computational cost is critical for its feasibility. Although the reaction-diffusion (monodomain) model remains the gold standard for arrhythmia simulations (Arevalo et al., 2016; Coleman et al., 2024), its high computational cost limits the scalability of the digital twin generation pipeline. Thus alternative simplifications have been proposed including i) the reaction-diffusion-Eikonal model (Neic et al., 2017), which uses the Eikonal model to speed-up the simulation of wave propagation, and ii) the Eikonal-type model coupled to an action potential shape model to recover transmembrane voltages, which does not include any representations of diffusion effects. In this study, we propose an open-source extension of the second approach by adding approximations of diffusion effects to achieve fast simulations of physiological repolarisation patterns and T-wave morphology. This allows us to perform tens of thousands of forward simulations for the inference process per subject.

## 2 Methods

**Figure 1:**
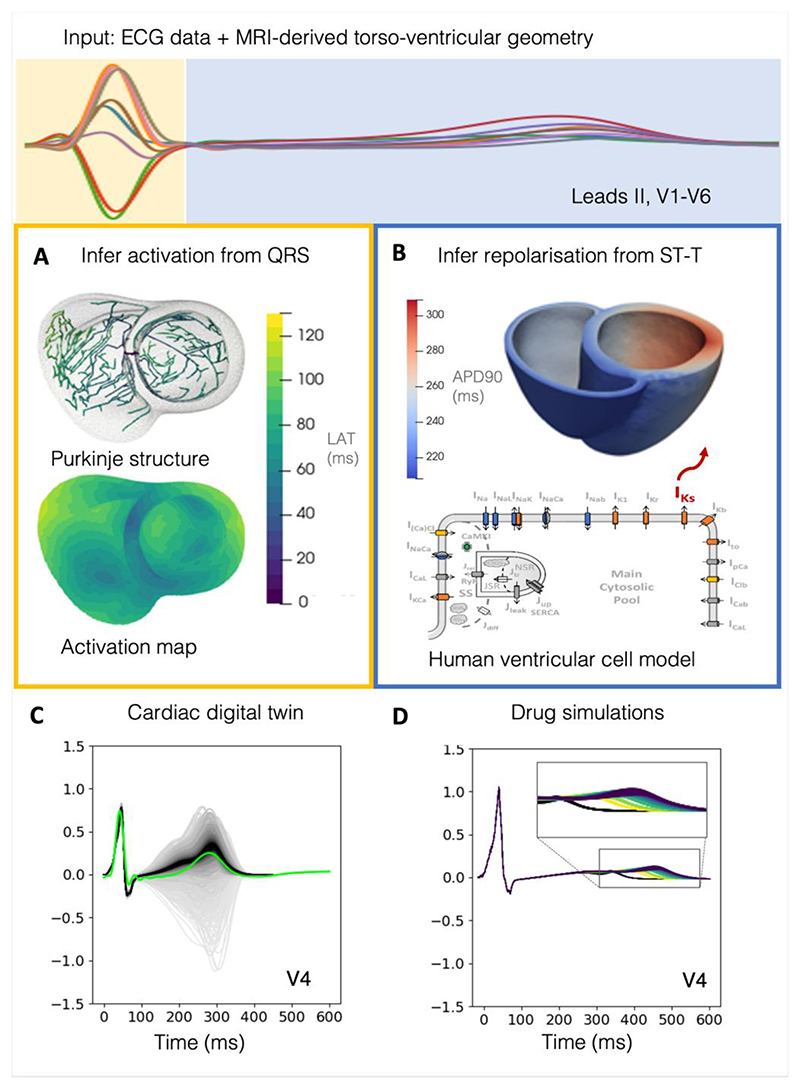
Overview of our cardiac digital twin personalisation pipeline. (**A**) Firstly, the pipeline infers the conduction speeds and the Purkinje-informed locations of earliest activation on the endocardial surface of the heart from matching QRS simulations to the subject’s ECG (Camps et al., 2024). (**B**) The pipeline then infers the spatial heterogeneity of the slow delayed rectifier potassium current (I_Ks_), which underpins repolarisation heterogeneity, by matching simulations of the ST segment and T wave to the subject’s ECG. (**C**) This process produces the cardiac digital twin, which contains the final inferred population of models (black traces) that matches the subject’s ECG (green trace). (**D**) These models are then translated to monodomain simulations, and Dofetilide application is added to evaluate the ability of the model to accurately predict drug effects, where the black traces are the baseline and the coloured traces are at varying doses of Dofetilide application.

Our pipeline builds on components for personalising ventricular anatomy (Zacur et al., 2017; Banerjee et al., 2021) and activation properties (i.e., Purkinje structure and conduction speeds) from the QRS segment of the ECG (Fig. 1.A) (Camps et al., 2024), and the novelty lies in the integration of algorithms and code to infer repolarisation from the ST segment and T wave of the ECG (Fig. 1.B), using a fast pseudo-diffusion reaction-Eikonal model. Our pipeline allows uncertainty propagation (Fig. 1.C) for predictions of therapy responses through personalised monodomain simulations, which we demonstrated by predicting the effect of various doses of Dofetilide (Fig. 1.D) and comparing predicted outcomes to clinical evidence (Johannesen et al., 2014; Vicente et al., 2015).

### 2.1 Data preparation and field generation

This study used clinical MRI and 12-lead ECG data from three healthy subjects (Table 1). This subject cohort included participants with different age, sex, body shape, heart size, and resting heart rate to test the robustness of our pipeline. Ventricular meshes were generated from the MRI data at ~1.5 mm and ~0.25 mm for pseudo-diffusion reaction-Eikonal and monodomain simulations, respectively (details can be found in Appendix A.1). Electrode positions were informed by rule-based locations on a segmented torso geometry (Appendix A.1).

the biventricular geometries, we generated rule-based descriptions of the fibre, sheet, and sheet-normal vector fields (Doste et al., 2019). We also generated four ventricular coordinates including the apex-to-base coordinate *ab(**x**)* and rotational *rt(**x**)* as defined by Cobiveco (Schuler et al., 2021), the transmural coordinate *tm(**x**)* as in Bayer et al., (2018), and the transventricular coordinate (left-to-right ventricle) *tv(**x**)* and the posterior-to-anterior *pa(**x**)* coordinates by normalising the projections of the nodal coordinates along the transventricular and posterior-to-anterior directions. These directions were defined as the normal to the septal surface (transventricular) and the cross product of the septal surface and basal surfaces normals (posterior-to-anterior) (Doste et al., 2019).

### 2.2 Decoupling Reaction and Diffusion for fast electrophysiological simulations

We propose a pseudo-diffusion reaction-Eikonal method to achieve faster simulation of ventricular electrophysiology than the existing reaction-diffusion models. The monodomain model (Potse et al., 2006) for simulating cardiac electrophysiology can be written as

(1) $$\frac{\partial U}{\partial t}=\frac{1}{{\text{C}}_{\text{m}}\chi }\left\{\nabla_{x}\cdot \left(\sigma \nabla_{x}U\right)-\chi I_{ion}(U,w,c)-\chi I_{app}(x,t)\right\},{\text{Ω}}_{0}\times (0,T]$$

where *χ* is the cardiomyocyte surface-to-volume ratio, *C_m_* is the membrane capacitance per unit area of the average cardiomyocyte, *U* is the membrane potential, *I_ion_* are the sum of ionic currents from the cell model where ***w*** and ***c*** are the ionic channel gating variables and intracellular ionic concentrations of the model, *I_app_* is the stimulus current applied at 3D ventricular scale, and **σ** is the orthotropic tensor of local conductivities in the reference configuration defined as,

(2) $$\sigma =\sigma_{f}f⊗f+\sigma_{s}s⊗s+\sigma_{n}n⊗n,$$

where σ_*f*_, σ*_s_*, σ*_n_* are the conductivities in the fibre ***f***, sheet ***s***, and normal ***n*** directions, respectively.

A well-accepted method for solving this equation is to perform time integration with an operator splitting approach. Eq. (1) is treated as the successive action of a reaction operator (*A*) and a diffusion operator (*B*):

(3) $$A:\frac{\partial U}{\partial t}=-\frac{1}{{\text{C}}_{\text{m}}}\left(I_{ion}-I_{app}\right),$$

(4) $$B:\frac{\partial U}{\partial t}=\frac{1}{{\text{C}}_{\text{m}}\chi }\nabla_{x}\cdot \left(\sigma \nabla_{x}U\right),$$

where the reaction operator *A* (Eq. 3) and the diffusion operator *B* (Eq. 4) are solved sequentially within each time step. An alternative to this is the reaction-diffusion-Eikonal model, proposed by Neic et al., (2017), where the activation wavefront (*t_a_*(***x***)) is approximated using the solution of an Eikonal model,

(5) $$\{\begin{array}{c} \sqrt{\nabla t_{a}^{T}(x)V\nabla t_{a}(x)}=1in{\text{Ω}}_{x}\\ t_{a}(x)=t_{i}forx=y_{i},wherei=1\ldots N_{root} \end{array}$$

where ***V*** is the conduction velocity tensor (prescribing orthotropic conduction in the fibre, sheet, and normal directions), and *t_i_* is the activation time of the *N_root_* earliest activation root nodes located at *y_i_*. The activation wavefront then provides the timing of the application of a stimulus current (*I_foot_*(***x***, *t_a_*)):

(6) $$I_{foot}(x,t)=\{\begin{array}{c} I_{foot}(t),ift\in \left[t_{a}(x)-T_{foot},t_{a}(x)\right]\\ 0,otherwise \end{array},$$

which is added as an additional stimulus current when solving the reaction-diffusion-Eikonal system:

(7) $$\frac{\partial U}{\partial t}=\frac{1}{{\text{C}}_{\text{m}}\chi }\left\{\nabla_{x}\cdot \left(\sigma \nabla_{x}U\right)-\chi I_{ion}(U,w,c)-\chi I_{foot}(x,t)\right\},{\text{Ω}}_{0}\times (0,T].$$

The reaction-diffusion-Eikonal model is equivalent to the monodomain model with the added advantage that it lifts its constraint of high spatial resolution (Neic et al., 2017). A simplification of this model is the no-diffusion-reaction-Eikonal formulation, where the diffusion operator *B* is ignored (Eq. 4):

(8) $$\frac{\partial U}{\partial t}=\frac{1}{{\text{C}}_{\text{m}}\chi }\left\{-\chi I_{ion}(U,w,c)-\chi I_{foot}(x,t)\right\},{\text{Ω}}_{0}\times (0,T].$$

The no-diffusion-reaction-Eikonal formulation is computationally beneficial as it contains only purely local pointwise operations (Neic et al., 2017). However, this formulation yields sharp changes in membrane potential and negatively impacts the T wave morphology in the ECG.

Here we propose a pseudo-diffusion reaction-Eikonal model that further reduces the computational cost compared with the reaction-diffusion-Eikonal model in Eq. (7), while including an approximation of diffusive repolarisation effects to allow smooth simulations of repolarisation and physiological T waves. To do this, we begin with the no-diffusion-reaction-Eikonal model (Eq. 8), and make the following modifications and additions:

1. We replace the fast-marching method used to solve the Eikonal model in Neic et al., (2017) by the faster Dijkstra algorithm (Wallman et al., 2012; Camps et al., 2021).
2. We replace the *I_foot_* current in the original no-diffusion-reaction-Eikonal formulation, which lasts for 1 ms, with a diffusive current (Gassa et al., 2021) that lasts for the duration of the activation phase of the cardiac cycle (Section 2.2.1),
3. We use the human biophysically-detailed ventricular cellular ToR-ORd model to describe ionic currents and create a precomputed look-up table of cellular action potentials for faster computation of the reaction operator A (Section 2.2.2).
4. We use smoothing to approximate the diffusion operator B during the repolarisation phase of the cardiac cycle (Section 2.2.3).

With these modifications, our pseudo-diffusion reaction-Eikonal model is essentially performing a ‘full split’ of the operators *A* and *B* (Eqs. 3 and 4), where we first solve *A* for the entire cardiac cycle and then apply *B* to that solution. This holds some similarities and differences with standard splitting techniques for the solution of the monodomain equation. If *e* ^*A*Δ*t*^ and *e*^*B*Δ*t*^ denote the exact solutions to subproblems Eq. (3) and Eq. (4), respectively, then these can be combined in each successive time step Δ*t* to yield the solution of the full monodomain equation by means of standard Euler or Godunov splitting techniques (Bueno-Orovio et al., 2006; Vigmond et al., 2008; Sachetto Oliveira et al., 2018) as:

(9) $$U(t+\text{Δ}t)=e^{A\text{Δ}t}e^{B\text{Δ}t}U(t)+O(\text{Δ}t).$$

Dropping the error term, and successively applying these operators in each time step, it implies that the solution to the reaction-diffusion equation at any arbitrary time step can be approximated as:

(10) $$U(n\cdot \text{Δ}t)\approx {\left(e^{B\text{Δ}t}e^{A\text{Δ}t}\right)}^{n}U(0),$$

where *n* is the number of time increments, and *U* (0) the initial condition. We can rewrite Eq. (8), as:

(11) $$\hat{U}(n\cdot \text{Δ}t)={\left(e^{A\text{Δ}t}\right)}^{n}U(0),$$

where $\hat{U}$ is equivalent to the no-diffusion-reaction-Eikonal solution in Eq. (8). Thus, under the assumption that operators *A* and *B* commute, the full solution given by Eq. (10) can be approximated as:

(12) $$U(n\cdot \Delta t)\approx {\left(e^{B\Delta t}\right)}^{n}\hat{U}(n\cdot \Delta t).$$

We adopted this formulation (Eq. 12) and solved first the reaction operator *A* before applying the diffusion operator *B* to its solution. Hereafter we describe how we solve Eq. (12).

#### 2.2.1 Using the diffusive current as stimulus for the reaction operator during activation

Firstly, to solve operator *A* (Eq. 3), we applied a stimulus current that was equal to the median diffusive current *I_diff_* during the activation phase of the cardiac cycle (first 100 ms),

(13) $$I_{diff}=\nabla_{x}\cdot \left(\sigma \nabla_{x}U\right)fort=[0,100]ms$$

such that operator A became:

(14) $$A:\frac{\partial U}{\partial t}=-\frac{1}{C_{m}}\left(I_{ion}-I_{diff}\right)$$

Following Gassa et al., (2021), our diffusive current was parameterised using the sum of two Gaussian functions:

(15) $$I_{diff}=A_{1}e^{-\frac{{\left(t-\mu_{1}\right)}^{2}}{2\sigma_{1}^{2}}}-A_{2}e^{-\frac{{\left(t-\mu_{2}\right)}^{2}}{2\sigma_{2}^{2}}},$$

where *A*_1_and *A*_2_ are magnitudes, *μ*_1_ and *μ*_2_ are times representing the Gaussian bump centers and *σ*_1_and *σ*_2_ are the standard deviation of the Gaussian functions.

It is worth noting that here *I_diff_* performs a different function to *I_foot_* in Neic et al., (2017), which only lasts for ~1 ms. In the reaction-diffusion-Eikonal model, the diffusion operator *B* was solved alongside operator *A* at every time step, and *I_foot_* was only necessary to approximate the effect of the arrival of the activation wavefront.

However, in the pseudo-diffusion reaction-Eikonal model, we are ignoring the diffusion operator *B* entirely when solving operator *A*.Thus, we instead use *I_diff_* (Eq. 15) to stimulate the action potentials in our pseudo-diffusion reaction-Eikonal model. This allows taking into account the effect of the currents that would activate the cellular models in a monodomain simulation.

We extracted *I_diff_* from a 3D biventricular (Subject 2 at 0.25 mm resolution) monodomain simulation using the MonoAlg3D solver (Sachetto Oliveira et al., 2018). The conductivities were calibrated to the subject’s QRS segment (Camps et al., 2024) and for the electrophysiology we used the ToR-ORd model. We took the median diffusive current and fitted the parameters in Eq. (15) to get a parameterised *I_diff_*.

#### 2.2.2 Precompute look-up table of human ventricular action potentials

The solution to the reaction operator *A* produces local action potentials that are activated as the Eikonal wavefront propagates through the spatial domain. The action potentials are parameterised using the local action potential duration at 90% repolarisation (APD), which is a spatially varying field (*APD(**x**)*), such that the reaction operator A becomes:

(16) $$A:\frac{\partial U}{\partial t}=-\frac{1}{C_{m}}\left(I_{ion}(APD(x))-I_{diff}\right)$$

To ensure fast simulation, we precomputed a look-up table of action potentials using the ToR-ORd model of human ionic current dynamics, which has been extensively validated using experimental data for disease phenotypes and drug response (Tomek et al., 2019; Zhou et al., 2022). This means that we no longer need to compute ionic current dynamics on the fly, which makes the computational cost tractable. Previous studies have explored using shape models with only a handful of parameters to approximate cellular action potentials, and the simplicity of these models allows fast simulation (Pezzuto et al., 2017; Gillette et al., 2021); however, these shape models have insufficient biophysical detail for drug simulations.

Spatial variations in various ionic currents underpinning repolarisation heterogeneity have been documented in the literature in both canine and human data, as summarised in Table A.1. From this table, the most robust evidence for spatial heterogeneity in the transmural, apex-to-base, and transventricular directions is for the slow delayed rectifier potassium current (I_Ks_) and the transient outward potassium current (I_to_). We selected only the G_Ks_ conductance to modulate APD because it has a larger effect on the APD than G_to_, and also selecting only one conductance preserves a one-to-one correspondence of APD to conductance value in the look-up table.

We uniformly sampled the conductance of G_Ks_ between 1/50 to 50-fold its baseline value, and applied the parameterised diffusive current *I_diff_* as stimulus (Eq. 14) to create a population of models (Britton et al., 2013), which was then calibrated to experimental ranges (online supplement, Table E.1) (Passini et al., 2019). We calculated the APDs of this calibrated population and used them to create a look-up table *K_APD_* of action potentials:

(17) $$K_{APD}:APD\to \left[G_{Ks};U_{APD}(t)\right],$$

where a given APD value is mapped to its corresponding G_Ks_ and a simulated action potential *U_APD_*. Coupling these precomputed action potentials (Eq. 17) with the Eikonal model’s wavefront (Eq. 5) and a spatially varying APD field (*APD(**x**)*) gives the solution to the reaction operator *A* (Eq. 11) as:

(18) $$\hat{U}(x,t)=\{\begin{array}{c} U_{rest},whent<t_{a}\\ U_{APD(x)}\left(t-t_{a}(x)\right),whent\geq t_{a} \end{array},$$

where *U_rest_* is the resting membrane potential before the action potential’s upstroke, and the timing of electrical activation *t_a_* (***x***) comes from the solution to the Eikonal model (Eq. 5).

The baseline ToR-ORd model was modified in order to achieve a sufficiently large range of APDs through varying G_Ks_ alone. To do this, we increased the baseline G_Ks_ by 5-fold (Doste et al., 2022), reduced the conductance of the rapidly activating delayed rectifier potassium current (G_Kr_) by 50%, and decreased the time constant of L-type calcium channel activation (*τ_jca_*) from 75 to 60 ms (Tomek et al., 2020). Each cellular simulation was repeated for 100 beats to achieve steady state, using a personalised cycle length calculated from the ECG heart rate, and the resulting action potentials *U_APD_* were saved for 600 ms at 1000 Hz. The upper and lower bounds of the simulated APD were set to equal the duration of the ST segment and the QT interval for each subject, respectively. Therefore, the shape of the lookup table *K* was *N*×(600 + 1), for a total of N integer values of APD in the prescribed range.

#### 2.2.3 Use smoothing to approximate the diffusion operator during repolarisation

After we have solved the reaction operator *A* (Eq. 16), we then apply a smoothing function (*S*) to its solution $\left(\hat{U}(x,t)\right)$ to approximation the diffusion operator *B* (Eq. 4) during repolarisation. Eq. (12) then becomes:

(19) $$U(n\cdot \text{Δ}t)\approx {\left(e^{B\text{Δ}t}\right)}^{n}\hat{U}(n\cdot \text{Δ}t)={\left[S(\hat{U})\right]}^{n}.$$

We can further reduce the computation of the pseudo-diffusion reaction-Eikonal simulations by considering different values for Δ*t* when solving operators *A* and *B*. Thus, we can rewrite Eq. (19) as

(20) $$U(t)\approx {\left(e^{B\Delta t_{B}}\right)}^{n_{B}}\hat{U}(n_{A}\cdot \Delta t_{A})={\left[S(\hat{U})\right]}^{n_{B}},$$

where Δ*t_A_* and Δ*t_B_* are the time steps for solving operators *A* and *B*, respectively, and *n_A_* and *n_B_* are the number of times that they need to be applied to simulate time *t*, namely, *t* = *n* · Δ*t*. Thus, the smoothing operator *S* is repeated *n_B_* times at the *n_B_* -th time step.

Our proposed smoothing operator was applied for each node in the solution $\left({\hat{U}}_{i}\right)$ using a weighted average of the membrane potential of all its adjacent nodes $\left({\hat{U}}_{m}\right)$, as well as of itself, and takes the form:

(21) $$S(\hat{U})=\sum_{m=1}^{M}\left(\frac{k_{m}{\hat{U}}_{m}}{\sum_{m=1}^{M}k_{m}+k_{i}}\right)+\frac{k_{i}{\hat{U}}_{i}}{\sum_{m=1}^{M}k_{m}+k_{i}},$$

where *M* is the total number of neighbours per node, and an adjacency weighting factor (*k_m_*) was designed such that the edge vector most aligned with the direction of the highest conduction speed has the highest weighting. Let the edge vector between a node and its adjacent node be ***p***, with ‖***p***‖ the distance to the adjacent node. The adjacency weighting factor *k_m_* is then:

(22) $$k_{m}=\frac{1}{\sqrt{{\left(\frac{\max(V_{f},V_{s},V_{n})}{V_{f}}p\cdot f\right)}^{2}+{\left(\frac{\max(V_{f},V_{s},V_{n})}{V_{s}}p\cdot s\right)}^{2}+{\left(\frac{\max(V_{f},V_{s},V_{n})}{V_{n}}p\cdot n\right)}^{2}}},$$

where *V_f_,V_s_,V_n_* are the conduction speeds along the fibre ***f***, sheet ***s***, and normal ***n*** directions, respectively. Note that *k_m_* is bound by

(23) $$\frac{\min\left({\text{V}}_{\text{f}},V_{s},V_{n}\right)}{\max\left(V_{f},V_{s},V_{n}\right)}\frac{1}{\left\|p\right\|}\leq k_{m}\leq \frac{1}{\left\|p\right\|}.$$

The membrane potential from the current node is weighted by a self-weighting factor *k_i_*, where *k*^−1^ is equivalent to the distance to itself in the weighting.

For this study, we calibrate *k_i_* and Δ*t_B_* in the pseudo-diffusion reaction-Eikonal model to match the repolarisation pattern of a corresponding monodomain simulation of Subject 2 (*k_i_* = 10 *mm*^−1^ and Δ*t_B_* = 20 *ms*). We applied the smoothing between the approximate end of activation (100 ms) and the end of repolarisation (450 ms). These times were extracted from the clinical ECGs across our three subjects.

#### 2.2.4 Action potential duration field

We have compiled predominantly human experimental evidence in the literature on spatial heterogeneities in APD (Table A.2). This showed APD spatial heterogeneities in both magnitude and direction of increase in the transmural, apex-to-base, transventricular, and posterior-to-anterior directions. Due to this variability in the available data, we allowed the spatial APD gradients to vary in direction and magnitude along these directions in the ventricles during the inference process.

**Figure 2:**
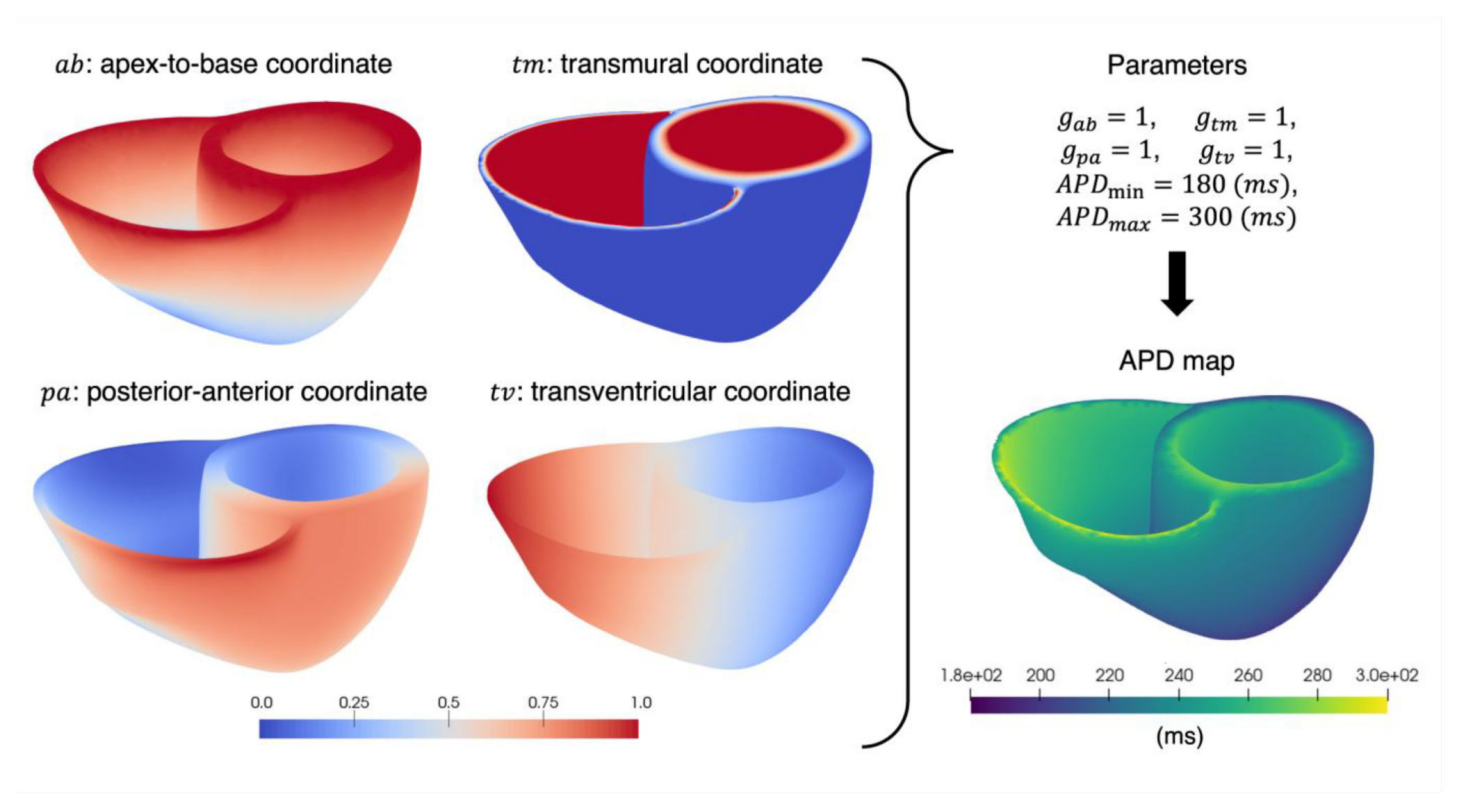
Biventricular coordinates were used to produce a representative APD map, which is generated using a linear combination of gradient weights (*g_ab_, g_tm_, g_pa_*, and *g_tv_*) along these coordinates with a specified APD range.

The *APD (**x**)* field is described as smoothly varying (Gillette et al., 2021), and is parameterised using a weighted linear sum of four ventricular coordinates, for which APD variations were reported in experimental studies (Table A.2). The chosen ventricular coordinates were the apex-to-base coordinate *ab (**x**)*, the transmural coordinate *tm (**x**)*, the transventricular coordinate (left-to-right ventricle) *tv (**x**)*, and the posterior-to-anterior *pa (**x**)* coordinate (Fig. 2). The APD field is then mapped to a physiological range of APDs specified by *APD_min_* and *APD_max_*, as follows:

(24) $$APD(x)=\left(\frac{q(x)-q{\left(x\right)}_{\min}}{q{\left(x\right)}_{\max}-q{\left(x\right)}_{\min}}\right)\left(APD_{\max}-APD_{\min}\right)+APD_{\min},$$

(25) $$q(x)=g_{ab}ab(x)+g_{tm}tm(x)+g_{pa}pa(x)+g_{tv}tv(x).$$

The weighting parameters *g_ab_, g_tm_, g_pa_*, and *g_tv_* control the relative magnitude of the APD gradient in their respective coordinate directions. At any point in the mesh, the relationship between local APD and the ionic conductance G_Ks_ is achieved through the look-up table *K_APD_* (Eq. 17), which means that a smoothly varying G_Ks_ field can be easily reconstructed from an APD field.

### 2.3 Electrocardiogram simulation

The pseudo-ECG (Gima & Rudy, 2002) method was used to simulate the 12-lead ECG. This method provides a fast and simple evaluation of the normalised ECG without major loss of morphological information compared with bidomain simulations (Wallman et al., 2012; Camps et al., 2021). Details on this can be found in Appendix A.2. The simulated 12-lead ECGs are normalised with respect to the R-progression of the clinical data (Camps et al., 2024).

### 2.4 Sensitivity analysis of ECG characteristics to APD gradients

Experimental evidence on APD gradients summarised in Table A.2 reveals variability in apex-to-base, posterior-to-anterior, transventricular, and transmural directions. Therefore, we aimed to explore the effect of these APD gradients on the simulated ST and T wave recordings using the pseudo-diffusion reaction-Eikonal model paired with the pseudo-ECG equation. We performed a global sensitivity analysis of these parameters in both positive and negative ranges on biomarkers extracted from the simulated ST segments and T waves. This sensitivity analysis was done using Subject 2’s anatomy, and the ranges for the *APD_min_* and *APD_max_* were calibrated based on their QT_onset and QT intervals. We defined the parameter ranges as [-1, 1] for the spatial gradients (*g_ab_ g_tm_ g_pa_ g_tv_*), and [180-230] ms and [270-300] ms, for the *APD_min_* and *APD_max_*, respectively (Sobol′, 2001), and the sampling was done using the Saltelli sampling (Saltelli, 2002; Saltelli et al., 2010), considering uniform priors for all parameters. The quantities of interest were the mean QT interval, T peak to T end interval, T wave amplitude, and T wave polarity across leads I, II, V1, V2, V3, V4, V5, and V6 using gradient-based evaluation methods. Total and first-order sensitivity effects were evaluated. Sampling and Sobol indices calculations for the sensitivity analysis were done using the SALib Python library (Herman & Usher, 2017; Iwanaga et al., 2022).

### 2.5 Inference of activation and repolarisation parameters

Following Camps et al., (2021), we have implemented the sequential Monte Carlo approximate Bayesian computation (SMC-ABC) algorithm to infer the activation and repolarisation parameters from clinical 12-lead ECG. This inference method allows iterative sampling of parameters and comparing the resulting simulations to the subject’s clinical ECG recordings until the population converges to a population that complies with a target cut-off discrepancy. An explanation of the SMC-ABC method and relevant hyperparameters can be found in Appendix A.3.

Compared to previous studies (Camps et al., 2021, 2024), the novelties in this study regarding the inference are the use of a different discrepancy metric (Section 2.5.2) and inferring repolarisation properties (Section 2.5.4).

#### 2.5.1 ECG discrepancy metric for inference from clinical QRS, ST and T wave signals

The dynamic time warping strategy proposed in Camps et al., (2021) was designed with a parameter space including conduction speeds. However, in this study we explore using a Pearson’s correlation coefficient-based (PCC) discrepancy metric that, while being less informative with respect to the parameter space, it will enable identifying local minima once the simulated and clinical ECGs have sufficient morphological similarities. This new discrepancy was defined as the weighted sum between squared inverse of the PCC and a normalised root mean squared error (RMSE). Thus, the discrepancy between a simulated and clinical ECG was defined as:

(27) $$\epsilon =\frac{1}{L}\sum_{i=0}^{L}{\text{w}}_{\text{p}}{\left(1-PCC_{i}\right)}^{2}+{\text{w}}_{\text{r}}\frac{RMSE_{i}}{\max\left(\left|R_{clinical}\right|\right)},$$

where *PCC_i_* is the Pearson’s correlation coefficient at lead *i*, evaluated between each simulated and clinical lead, then averaged over all leads (*L* = 8), *RMSE_i_* is the root mean squared error for each lead *i* between each simulated and clinical lead, then averaged over all leads, w_p_ and w_r_ are the weighting factors for the PCC and RMSE errors, and max(|*R_clinical_*|) is the maximum R wave amplitude across all leads of the normalised clinical ECG data. Note that the RMSE is dimensionless because it has been calculated based on normalised ECG signals. This discrepancy has the advantage that it will perform similarly regardless of the underlying parameter space as long as it modifies the morphology and amplitude of the 12-lead ECG. For this study, we set *w_p_* to 100, and *w_r_* in Eq. (27) to 2 to place greater importance on matching ECG morphology rather than absolute signal amplitude.

#### 2.5.2 Inference of conduction speeds and root nodes to match the QRS complex data

The biventricular electrical activation pattern is inferred from the 12-lead QRS ECG segment using the methods found in Camps et al., (2024). Briefly, the activation time of each root node was prescribed using a human-based physiologically-informed Purkinje tree network and inferred alongside the sheet *V_s_*, endocardial dense *V_d_*, and endocardial sparse *V_e_* conduction speeds. The fibre *V_f_*, sheet-normal *V_n_*, and Purkinje *V_p_* conduction speeds were prescribed to 65 cm/s, 48 cm/s (Taggart et al., 2000), and 300 cm/s (Durrer et al., 1970; Myerburg et al., 1972, 1978), respectively. We selected the best-match parameter set from the inferred population and fixed these parameters for the repolarisation inference below.

#### 2.5.3 Inference of APD gradients to match ST segment and T wave ECG signals

As for the repolarisation phase, we run the SMC-ABC algorithm to infer a set of parameters that determine ST segment and T wave ECG signals, namely, the APD ranges and spatial gradients: *g_ab_, g_tm_, g_pa_, g_tv_, APD_min_, APD_max_*. The activation sequence was fixed using to the parameter set with the lowest discrepancy from the inference of activation properties (Section 2.4.2).

The parameter space for the repolarisation gradients was defined as continuous. However, we know that similar parameter configurations can yield indistinguishable simulation results when evaluated. Thus, to speed up the inference process and promote convergence to different solutions in the final population, we discretised the parameter space. The *APD_min_* and *APD_max_* were discretised to a resolution of 2 ms, and the gradient parameters (*g_ab_, g_pa_, g_tm_*, and *g_tv_*) were discretised to a resolution of 0.1 gradient units.

The inference hyperparameters were set to 120 for the population size, to 50% to the sampling rate, to 0.5 for the target discrepancy cut-off, and 50% for the uniqueness threshold. This configuration of hyperparameters enabled a reasonable coverage of the parameter space during a rapidly converging inference process due to the high sampling rate. However, we still set the target discrepancy cut-off to a low value with the aim to stress test how well the inference process could match the clinical ECG for each subject. Nevertheless, the uniqueness threshold ensured that at least we would obtain a ‘physiological envelope’ with 64 unique parameter-sets in our digital twins. This variability of the inferred digital twins represents uncertainty from solving the inverse electrocardiographic problem given the subject’s ECG and biventricular anatomy. This uncertainty can be propagated to drug simulations by translating from pseudo-diffusion reaction-Eikonal to monodomain simulations. The values for all hyperparameters for the inference can be found in the code repository.

### 2.6 Translation from Reaction-Eikonal to Monodomain simulations

In addition to the diffusive and smoothing strategies, as previously described (Section 2.4.2), we calibrated the orthotropic conductivity parameters σ*_f_*, σ*_s_*, σ*_n_* in the monodomain model to achieve desired conduction velocities using a cable simulations, as in Costa et al., (2013). The translation of the activation properties was done as described in Camps & Berg et al., (2024) using the Shocker algorithm (Berg et al., 2023).

We extracted the G_Ks_ scaling factors that correspond to the fitted APD maps of the inferred population and embedded them in the monodomain simulations. Closest-point interpolation was used to translate from the coarser mesh (1.5 mm edge length) used for reaction-Eikonal simulations to the finer mesh (0.25 mm edge length) used for monodomain simulations.

### 2.7 Evaluation of digital twins using virtual drug simulations

We simulated the effect of Dofetilide, an I_Kr_ (i.e., hERG channel) blocker, and benchmarked the outcomes on ECG biomarkers with a Phase 1 randomized study on healthy adult participants (Vicente et al., 2015). To reduce the computational requirements for the virtual therapy evaluations, we randomly selected a subset of 10% of the parameter-sets (n = 12) from the physiological envelope (i.e., digital twin) inferred for each subject and simulated the monodomain ECGs at a resolution of 0.5 mm (edge-length). These downsampled digital twins were used in place of the full inferred physiological envelopes in the drug evaluations presented in this study.

Seven doses of Dofetilide, matching the same range of concentrations present in the clinical trial were simulated: 0.5 nM, 1 nM, 2 nM, 3 nM, 4 nM, 5 nM and 6 nM, which correspond to reducing the I_Kr_ by approximately 40%, 50%, 60%, 66%, 70%, 73% and 76%, respectively (Crumb et al., 2016). The magnitude of these conductance blocks was calculated using the Hill Equation

(28) $$G_{x}Block=\frac{1}{1+{\left(\frac{\left[Drug\right]}{IC_{50}}\right)}^{h}}.$$

Dofetilide is known for inducing QTc prolongation, primarily through an increase in late-stage repolarisation observed in the measurement T peak to T end. The QTc prolongation and T peak to T end were calculated from the simulated ECG, QTc was obtained using Fridericia’s correction (Phan et al., 2015) as was performed in the clinical trial for a direct comparison of outcomes.

(29) $$QTc=\frac{QT}{RR^{1/3}},$$

where *QT* is the QT interval in ms and *RR* is the R to R interval in seconds, such that when the heart rate is 60 beats/minute (i.e., 1 beat/second), the *QTc* and the *QT* are equivalent.

### 2.8 Metrics

Besides the discrepancy metric used for the inference (Eq. 27), we report our results using standard metrics used in similar studies to ease comparison and interpretability of our results. When evaluating ECG differences we report the Pearson’s correlation coefficient (PCC) since this is standard practice in the ECG inverse problem community, and the root mean squared error (RMSE) between the R-wave normalised recordings (Bear et al., 2018; Pezzuto et al., 2021). We considered a good match of the simulated and clinical ECG when the PCC is larger than 0.9 since this was the value achieved in previous inference studies with known ground truths (Camps et al., 2021; Pezzuto et al., 2021). A PCC > 0.9 implies that the morphological resemblance between both ECGs makes them clinically equivalent.

#### 2.9 Computational and Software

A comparison of computational times between monodomain, pseudo-diffusion reaction Eikonal, and literature reports for the reaction-Eikonal models can be found in Table A.3. Monodomain simulations were performed using MonoAlg3D (Sachetto Oliveira et al., 2018), a high-performance open-source GPU solver (available at https://github.com/rsachetto/MonoAlg3D_C), on the Polaris supercomputer provided by the Argonne Leadership Computing Facility (ALCF) using 8 cores and 1 GPU (NVIDIA A100). A resolution of 0.25 mm was used for the translation from pseudo-diffusion reaction-Eikonal to monodomain and a resolution of 0.5 mm was used for the drug simulations. Further details on the numerical scheme for solving the monodomain equation can be found in online supplement E.4. The inference of repolarisation properties required 18 hours of computation time on average on a desktop computer (see Table A.4 for details for all three subjects).

## 3 Results

### 3.1 Inference results using the reaction-Eikonal models

**Figure 3:**
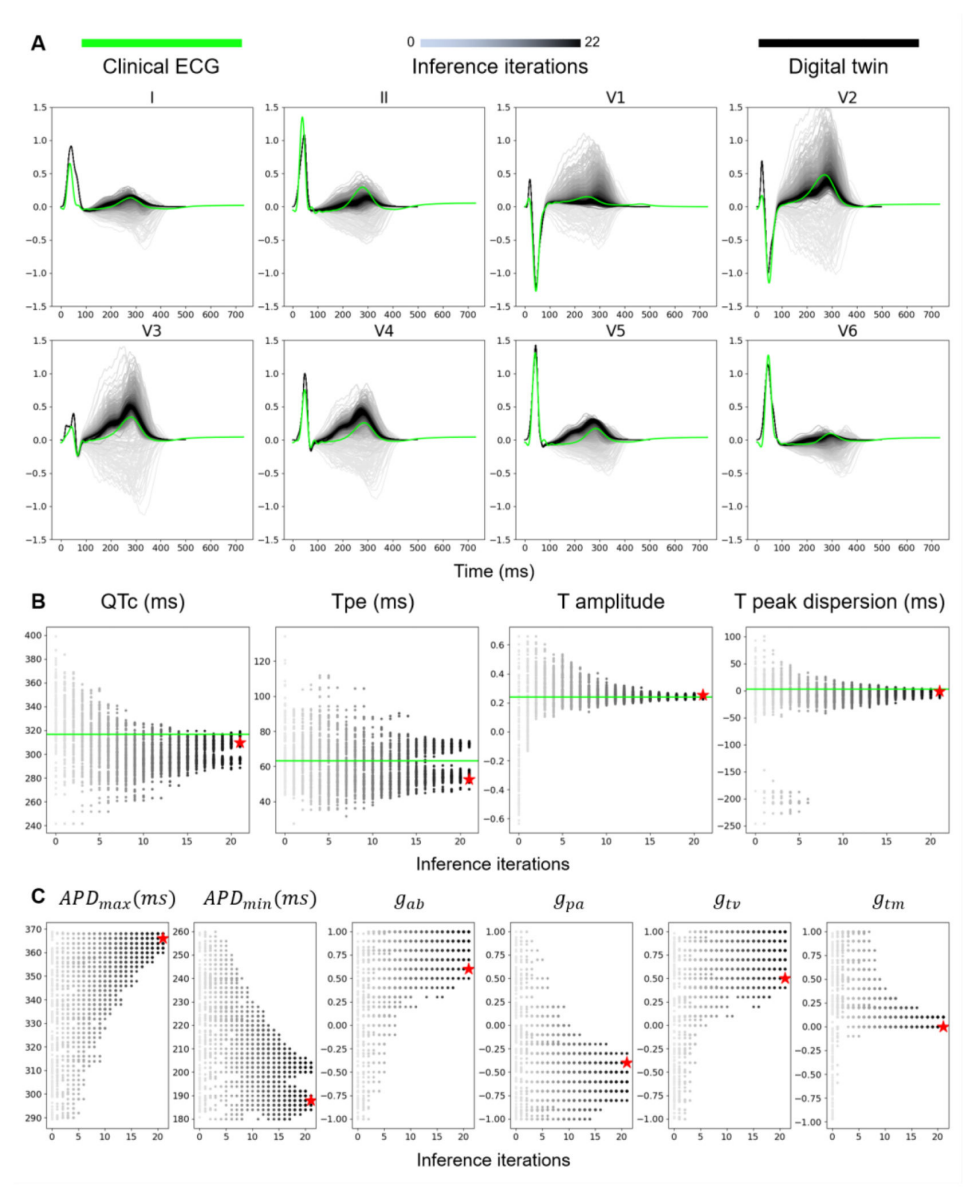
Inference (Subject 2) iterations effectively explore the T wave biomarker space. Clinical subject ECGs are shown in green, successive inference iterations are shown in decreasing luminance, and the ‘digital twin’ (final inferred population) is shown in black. A) Range of T wave morphologies explored by the inference process, converging to the population with the best match to clinical data. B) QT interval, T peak to T end interval (Tpe), average T wave amplitude, and dispersion of T peak timing between leads V3 and V5, converging over successive inference iterations (grey to black) to match clinical values (denoted by green horizontal lines). C) Progression of the parameter space over successive inference iterations. This parameter space was composed of *APD*_max_ (maximum action potential duration), *APD_min_* (minimum action potential duration), *g_ab_* (APD gradient in the apex-to-base direction), *g_pa_* (APD gradient in the posterior-to-anterior direction), *g_tv_* (APD gradient in the transventricular direction) and *g_tm_* (APD gradient in the transmural direction). The red stars in panels B and C indicate the parameter-set with the lowest discrepancy. Inference hyperparameter values: population size = 120, sampling rate = 50%, target discrepancy cut-off = 0.5, uniqueness termination threshold = 50%.

Fig. 3. illustrates the inference process for producing the cardiac digital twin of Subject 2. The simulated ECGs at each successive inference iteration is plotted in progressively darker shades of grey and the final physiological envelope of the digital twin is shown in black, together with the clinical ECG shown in green (Fig. 3.A). ECG biomarkers (Fig. 3.B) and model parameter values (Fig. 3.C) are plotted at each successive iteration and the model with the lowest discrepancy value is marked with a red star.

The inference process for Subject 2 (Fig. 3) explored a wide range of T wave biomarker values over 22 iterations (grayscale gradients in Fig. 3) before arriving at the final population. Interestingly, the T peak to T end (Tpe) biomarker converged into two different clusters of values (Fig. 3.B), while all other biomarkers seemed to have a single cluster of values in the final population. The parameter-set with the lowest discrepancy value (namely, the best-matched model) for Subject 2 was *APD_min_* = 216 (*ms*),*APD_max_* = 294 (*ms*),*g_ab_* = 1,*g_pa_* = −1,*g_tv_* = 0, *g_tm_* = 0 1 (red star in Fig. 3), which indicates significant apex-to-base (positive) and posterior-to-anterior (negative) gradients, with larger APD on the base than the apex, and on the posterior than the anterior. The effect of the transmural and transventricular gradients were less prominent.

The inferred population had a final discrepancy of 0.53 (Fig. 3). The inference process was terminated by the uniqueness threshold rather than by the discrepancy cut-off, meaning that the final population had less than 50% uniqueness in the parameter sets while the discrepancy was still above the desired cut-off of 0.5. This was a good outcome since it meant that the discrepancy cut-off of 0.5 was well-chosen to push the inference process to match the clinical data as well as possible while preserving the uniqueness of parameter sets in the population.

The digital twins of all three subjects were able to match both QRS and T wave morphologies in the clinical data (Fig. 3B, A.2, and A.3), achieving mean ± standard deviation Pearson’s correlation coefficient of 0.9±0.0006 and root mean squared error (RMSE) of 0.13±0.001 on average across all three subjects. This RMSE value was small, considering that the maximum amplitude of the QRS complex was ~1.5. The mean and standard deviation of the inferred parameter-sets for Subject 2 (Fig. 3.C) are reported in Table 4 alongside the inferred parameter-sets for Subjects 1 and 3. The inference progress for Subjects 1 and 3 are reported in Appendix A.7 (Figs. A.2 and A.3).

The simulated ECGs from the final population showed the presence of a bifid, or notched, T wave, which was likely due to a notched first derivative of phase three of the ToR-ORd cell model, which is reported in more detail in Appendix A.9.

### 3.2 Translation from reaction-Eikonal to monodomain simulations

We demonstrate the translation between pseudo-diffusion reaction-Eikonal to monodomain simulations using the parameter-set with the lowest discrepancy value (namely, the best-matched models) (red star in Figs. 3, A.2, and A.3) from the inferred population (‘physiological envelope’) for each subject.

**Figure 4:**
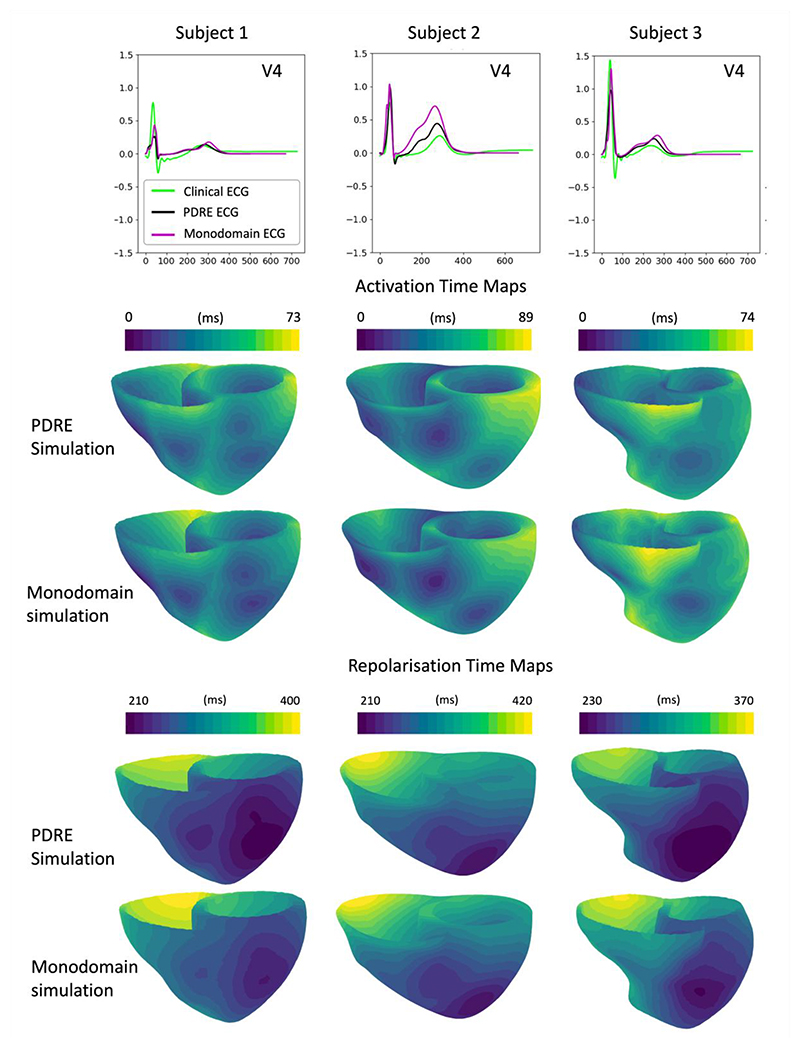
Translation of the inferred parameter-set with the lowest discrepancy for each subject (red stars in Fig. 3, Fig. A.1, Fig. A.2) from pseudo-diffusion reaction-Eikonal (PDRE) (at a resolution of 1.5 mm) to monodomain simulations (at a resolution of 0.25 mm). ECGs are shown for lead V4, followed by activation and repolarisation time maps.

The best-matched pseudo-diffusion reaction-Eikonal model was translated to monodomain simulations (at a resolution of 0.25 mm) by prescribing its spatial gradient of G_Ks_ scaling factors in the monodomain simulation, and calibrating the monodomain’s conductivity values (Eq. 2) to the inferred conduction speeds (Table A.5 and A.6). The translated monodomain simulation showed a good agreement with pseudo-diffusion reaction-Eikonal simulated ECG, and clinical ECG in terms of T wave morphology and polarity (Fig. 4). Activation and repolarisation maps were also well-matched in pattern between the pseudo-diffusion reaction-Eikonal and monodomain simulations (Fig. 4).

Translation between reaction-Eikonal and monodomain simulations was a key step in the realisation of virtual drug evaluations using cardiac digital twins. To match the conduction velocity characteristics of reaction-Eikonal and monodomain simulations, conductivities of the monodomain simulation (*σ_f_,σ_s_,σ_n_*) were calibrated using the TuneCV toolkit from MonoAlg3D (Tables A.5 and A.6).

**Figure 5:**
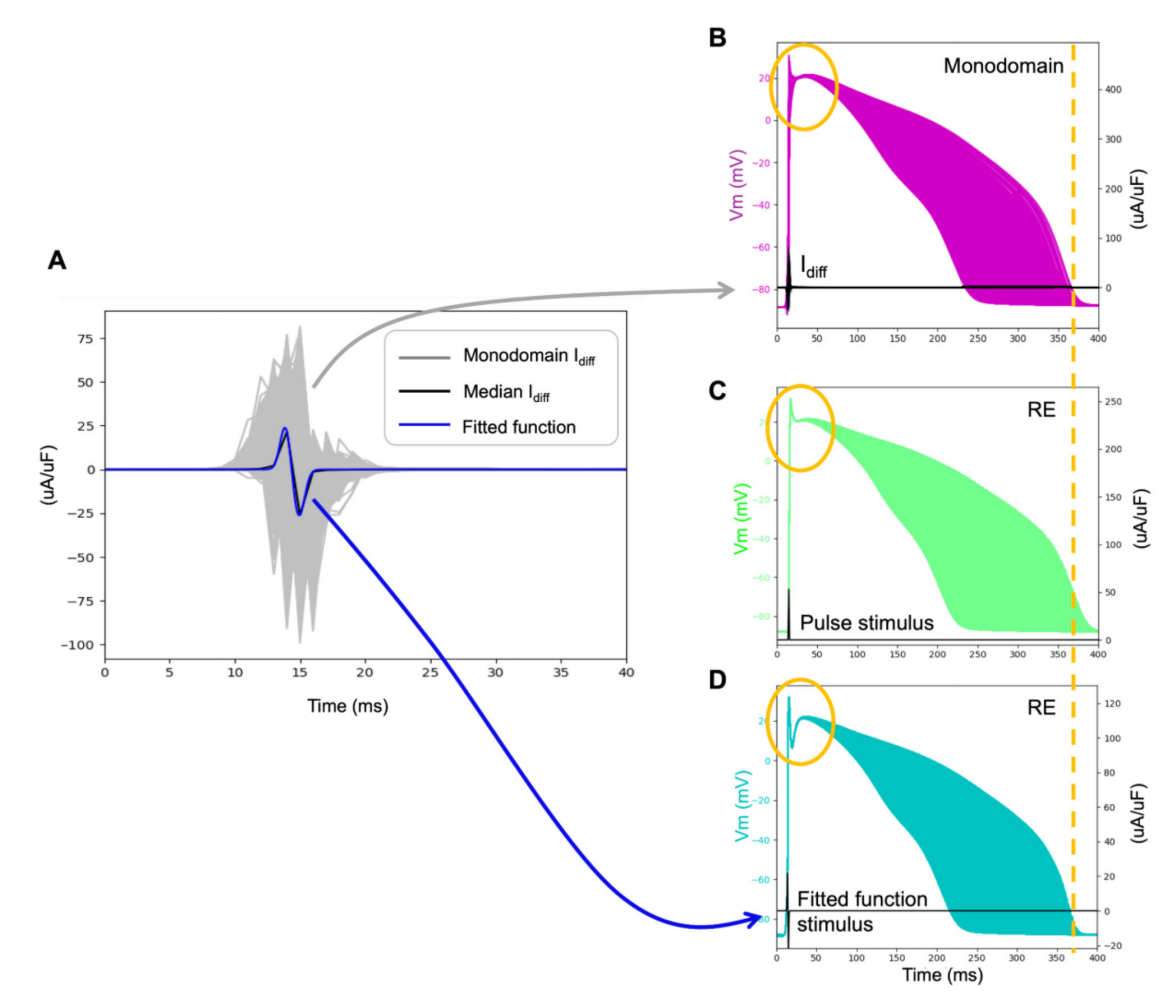
Diffusive current extraction. A) Diffusive currents extracted from monodomain simulation (Subject 2) (grey) with median (black) and fitted to Eq. (15) (blue) to obtain *I_diff_* (Eq. 14). B) Monodomain action potentials aligned using activation times. C) Reaction-Eikonal (RE) action potentials stimulated using a pulse stimulus, aligned using activation times. D) Reaction-Eikonal (RE) action potentials simulated using the fitted diffusive current *I_diff_* (as shown in panel A). The Reaction-Eikonal simulations were run without smoothing or diffusion.

The diffusive currents that were extracted (Fig. 5.A - grey traces) were predominantly biphasic (98.77%), with positive monophasic (1.2%) and negative monophasic (0.03%) curves in the minority. The parameters of Eq. (15) were fitted to the median diffusive current (Fig. 5.A - black trace) to produce the fitted function (Fig. 5.A - blue trace). The fitted parameters (Eq. 15) were A1=25.83 μA/ μF, A2=27.42 μA/ μF, μ1=13.9 ms, μ2=14.9 ms, σ1=0.406 ms, and σ2=0.4432 ms. The fitted diffusive current was used to stimulate the action potentials for the entire calibrated cell population of models (Fig. 5.D).

The inclusion of the diffusive stimulation current *I_diff_* (Eq. 14) resulted in a narrower range of APDs (Fig. 5.D compared to Fig. 5.C), which was also observed in the action potentials extracted from a monodomain simulation using the same ionic parameter values (Fig. 5.B). Our linear spatial gradient model (Eqs. 24, 25) imposes the extreme APD values (i.e., *APD_min_* and *APD_max_*) to the boundaries of the biventricular geometry. The monodomain is known to impact the APD at the boundaries by lengthening it in the stimulus regions and shorten it in distal locations (Bueno-Orovio et al., 2014). Thus, the disagreements in the APD ranges (Fig. 5.B vs Fig. 5.D) are likely to be produced by boundary effects in the monodomain simulation which are not incorporated in our pseudo-diffusion reaction-Eikonal model.

**Figure 6:**
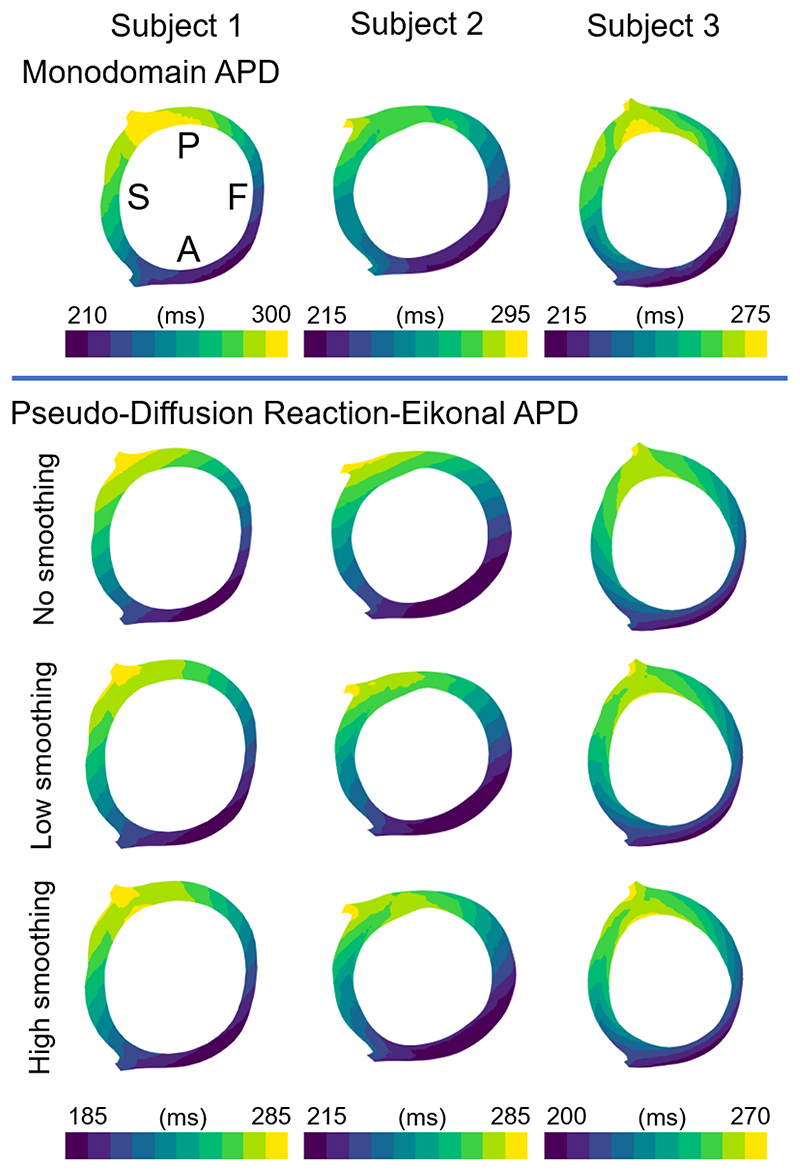
Comparison of effect of different amounts of smoothing on simulated action potential durations (APDs) of the pseudo-diffusion reaction-Eikonal (PDRE) to monodomain. First row shows monodomain simulations for the three subjects, showing a left ventricular short-axis slice (P – posterior, A – anterior, S – septum, F – freewall). Second, third, and fourth rows show the PDRE simulations with no smoothing, low smoothing (*k_i_* = 10 *mm*^−1^), and high smoothing (*k_i_* = 1 *mm*^−1^), respectively. The APDs calculated by subtracting the activation times to the simulated repolarisation time maps.

The smoothing self-weighting factor *k_i_* (Eq. 21) of the pseudo-diffusion reaction-Eikonal simulations dominates the strength of the diffusion without affecting the computation cost of the model. For the inference from all subjects, *k_i_* was set to 10 mm^-1^ (i.e., self-distance of 0.1 mm) (Section 2.2.3). Here (Fig. 6) we demonstrate the effect of this hyperparameter (*k_i_*) on the simulated activation and repolarisation time maps, using the configurations shown in Fig. 4 (i.e., best-matched models) as baseline (and keeping Δ*t_B_* as 20 ms).

The APDs shown in Fig. 6, which were calculated by subtracting the activation times from the repolarisation time maps, showed distinctly non-linear patterns in the monodomain simulations (Fig. 6 – first row), which were not recovered by the reaction-Eikonal simulations without orthotropic smoothing (Fig. 6 – second row). On the other hand, both values tested for *k_i_* (i.e., low smoothing – 10 mm^-1^ and high smoothing – 1 mm^-1^) were able to correctly reproduce these non-linear patterns similarly to the monodomain simulations (Fig. 6 – third and fourth rows). In summary, the orthotropic smoothing of the pseudo-diffusion reaction-Eikonal simulation improved the match to monodomain transmural repolarisation patterns across all three subjects. Moreover, the chosen value for *k_i_* was already able to recover most patterns in all three subjects, and further increasing it by tenfold yielded similar simulation results.

### 3.3 Virtual drug evaluations using a cardiac digital twin

**Figure 7:**
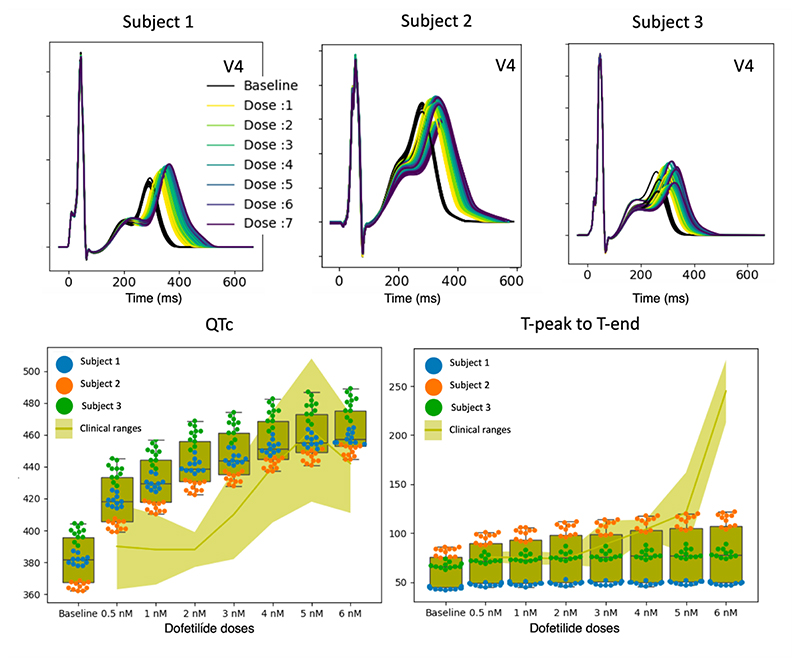
Dofetilide virtual evaluation using the monodomain digital twins (‘physiological envelopes’). Each subject’s digital twin was represented by a ‘physiological envelope’ including 12 inferred parameter-sets. Top row shows the effect of seven doses of dofetilide (dose 1: 0.5 nM, dose 2: 1 nM, dose 3: 2 nM, dose 4: 3 nM, dose 5: 4 nM, dose 6: 5 nM, dose 7: 6 nM) on the simulated ECG (lead V4) using the digital twins of the three subjects. Bottom row summarises effect of drug doses on corrected QT interval (QTc) and T peak to T end intervals of the digital twins compared with previously published clinical ranges (Vicente et al., 2015) since the data for the considered subjects was not available.

A 10% subset of the reaction-Eikonal models in the final inferred population (n=12) was randomly selected for simulations of the effects of the IKr (i.e., hERG channel) blocker Dofetilide (Fig. 7). These reaction-Eikonal models were translated into monodomain simulations to create a baseline population of ventricular electrophysiological models (black traces Fig. 7.A and 7.C). The simulated effect of Dofetilide application showed a graded increase in mean corrected QT interval (QTc) (Fig. 7.B, blue dots) and T peak to T end duration (Fig. 7.B, black dots), which matched the expected trend seen in clinical data (Johannesen et al., 2014; Vicente et al., 2015; Crumb et al., 2016) (Fig. 7.B, yellow and cyan traces, respectively). The steeper increase in T peak to T end duration in the clinical data compared with our simulations suggests spatially heterogeneous effects of the drug’s action that have not been accounted for in our modelling.

### 3.4 Global sensitivity analysis of T wave biomarkers to APD parameters using reaction-Eikonal models

Results of sensitivity analysis (Fig. A.3) performed using the reaction-Eikonal model showed strong positive correlations of QTc with maximum APD, T wave amplitude and polarity with the apex-to-base and transmural gradients. Global sensitivity analysis showed that maximum APD is by far the most important determinant of the QT interval, while the transmural and apex-to-base gradients were most important for T wave amplitude and polarity. T peak to T end duration was sensitive to all gradient parameters, with the transmural gradient having the largest effect by a small margin. Our analysis highlights the non-specificity of the T peak to T end duration in the ECG to repolarisation heterogeneity in any particular ventricular spatial axis.

## 4 Discussion

We present an open-source fast digital twinning pipeline from MRI and ECG data for multi-scale drug and disease investigations with uncertainty propagation. Key novelties of this pipeline are i) personalised repolarisation heterogeneities, which were enabled by ii) a pseudo-diffusion reaction-Eikonal model designed to capture human ventricular electrophysiology with low computational cost, for iii) personalised drug evaluations with uncertainty propagation demonstrated using Dofetilide.

### 4.1 Cardiac digital twins as a tool for tailored treatment planning and *in silico* trials

#### Uncertainty propagation is important for applications of the cardiac digital twin

When surveying methodological approaches for personalising cardiac electrophysiology, the ECG imaging approach is a well-established approach that has been extensively validated (Ramanathan et al., 2004; Andrews Christopher M. et al., 2017; Bear et al., 2019). However, the method relies on large numbers (n≈200) of body surface electrode recordings that allow the inverse problem to be solved using regularisation methods (Cluitmans et al., 2015; Bear et al., 2019). When considering only the standard 12-lead ECG more commonly found at the clinical setting, it becomes necessary to constrain the inference solution space and contend with increased levels of uncertainty (Giffard-Roisin et al., 2017; Camps et al., 2021; Pezzuto et al., 2021; Gillette et al., 2021). The Bayesian-based inference method in this study allows effective explorations of the parameter set space and effective propagation of uncertainties to any applications of the digital twin, such as for drug evaluations. Our cardiac digital twin is not a single personalised model but a spectrum of models that all fall within a ‘physiological envelope’ of ECG characteristics (Fig. 3). This implicitly allows uncertainty to be represented in the digital twin and propagated to drug evaluations, where individual models within the ‘physiological envelope’ can have varying responses.

Other sources of uncertainty can arise, such as from data measurement errors in the ECG signal (Bear et al., 2018, 2021), respiration motion effects, beat-to-beat variability, and uncertainty in electrode placements (Multerer & Pezzuto, 2021), and MRI segmentation uncertainties (Tate et al., 2021). However, even if the nature of variation in these sources of uncertainty across the population could be statistically modelled, it is difficult to predict how their interactions would manifest in the ECG. By using an approximate Bayesian computation approach (Camps et al., 2021), we do not need to specify the error structure and the statistical likelihood is approximated by a simpler notion of closeness to the clinical data. Specifying a target discrepancy threshold then amounts to specifying an amount of overall error, regardless of its source, from which point uncertainty quantification proceeds.

#### Biophysical detail in the digital twin is necessary for evaluating therapy and disease

Recent advances towards creating electrophysiological cardiac digital twins (Gillette et al., 2021) rely on the Mitchell-Schaeffer (Mitchell & Schaeffer, 2003) shape model of cellular action potentials, which lacks sufficient biophysical detail to accurately and meaningfully represent ionic channel dynamics, and therefore cannot be used for multiscale investigations into drug and disease effects. The need to explicitly represent different ionic channel dynamics similarly precludes the use of other minimal models of action potentials (Bueno-Orovio et al., 2008). In this study, we chose to use the ToR-ORd model of human electrophysiology given its extensive validation (Zhou & Wang et al., 2024). The trade-off between model usefulness with computational cost is resolved in our method by using a look-up table, which requires a judicial choice for the ranges of APDs to be included in order to allow full freedom of exploration of the parameter space during inference. Furthermore, to ensure that the cardiac digital twin can be used to quantify arrhythmic vulnerability of drug and disease effects in future studies, we have taken care to enable ease of translation between the pseudo-diffusion reaction-Eikonal model used in digital twin generation with monodomain simulations, which remain the gold standard for arrhythmia risk evaluation (Arevalo et al., 2016; Coleman et al., 2024).

#### Computational cost and scalability

While the reaction-diffusion-Eikonal formulation presented by Neic et al., (2017) presents a significant advance towards speeding up the forward simulation, using a reported 197 seconds per simulation (Gillette et al., 2021), a further speed up is necessary to enable the ~10,000 forward simulations required to fully explore the parameter space in the inference process. The no-diffusion-reaction-Eikonal formulation in Neic et al., (2017) has a simulation time of 8 seconds, which is much more suitable for the inference process. However, it suffers from non-smooth repolarisation, which limits its applicability for digital twin generation. Our pseudo-diffusion reaction-Eikonal model, built as an improve to the no-diffusion-reaction-Eikonal model by Neic et al., (2017) uses several strategies to achieve a faster simulation time of ~6 seconds per forward simulation while preserving smooth repolarisations. These considerations improve the scalability of the digital twin framework while preserving its relevance to therapy evaluation.

#### Generalisation to the variability in the human population

To ensure generalisation to the variability in the human population, we considered variabilities in anatomy and heart rate by demonstrating our pipeline in a cohort of three subjects with differences in age (23-76 years old), sex (2 female and 1 male), body mass index (21-34), heart rate (48-74), and myocardial mass (58-109 mm^3^). Our pipeline was able to automatically generate the digital twins for all three subjects without any manual modifications and to conduct virtual drug evaluations on them. However, there are additional considerations, such as sex and age differences in cardiac electrophysiology, which should be considered.

### 4.2 Impact of methodological choices

#### Sequential inference of activation and repolarisation characteristics

The presented pipeline, as well as similar studies (Gillette et al., 2021), assumes that the inference of repolarisation properties can be successfully addressed after inferring the activation properties. This assumption reduces the parameter space considerably, thus reducing the computational cost and complexity of the inverse problem while still enabling the finding of plausible solutions with a good match to the clinical data (Fig. 3). However, given sufficient resources, inferring both activation and repolarisation characteristics simultaneously could widen the functional variability of the inferred population. While this alternative may be non-scalable given the current state of high-performance computing resources, our pipeline has been equipped with the capability of conducting simultaneous inference on activation and repolarisation parameters.

#### Rule-based electrode locations

Electrode locations are known to impact the ECG (Mincholé et al., 2019; Multerer & Pezzuto, 2021). However, their locations are not registered during the acquisition of 12-lead ECG, thus, their location becomes a source of uncertainty when addressing the electrocardiographic inverse problem using these data. We have employed a deterministic rule-based strategy based on the reconstruction of the subject’s torso from MRI to position the electrodes used to simulate the 12-lead ECG. Errors in our electrode placement will impact how well we can match the clinical recordings, which may have limited the match achieved in this study. However, body surface potentials that are contiguous in space are likely to be similar to each other. Thus, by matching morphological features of the ECG, we can still generate meaningful digital twins.

#### Single ionic conductance (GKs) to drive APD heterogeneity

Experimental evidence identifies the I_Ks_ and I_to_ currents as the key ones responsible for APD heterogeneity (Table A.1). In our cellular simulations, the IKs current had a much larger effect on the APD than I_to_; thus, prompting our decision to vary only I_Ks_ in the whole ventricular simulations. In addition, since the I_to_ current has a significant effect on the morphology of phase one of the action potential and, therefore, on the QRS morphology, any future explorations of spatial variation in I_to_ would need to infer both activation and repolarisation characteristics simultaneously. Other morphological aspects of the action potential, such as APD50 and the plateau amplitude, and its influence on T wave morphology should also be investigated in future improvements, especially if multiple current heterogeneities were to be introduced.

#### Parameterisation of APD gradients

We reviewed experimental evidence and decided to consider linear APD gradients because the available data has insufficient spatial resolutions to justify and parameterise higher-order and/or non-monotonic gradients. Exponential-like gradients in the APD could help to allow greater regional heterogeneity in APD, which could be more relevant for disease phenotypes, such as in myocardial infarction (Zhou & Wang et al., 2024). Future studies could also consider the effect of the autonomic nervous system on altering the APD (Conrath & Opthof, 2006).

#### Stationary anatomy and other anatomical considerations

The T wave in the ECG occurs during the onset of ventricular contraction. The deformation in the ventricles is known to influence the shape of the T wave (Moss et al., 2021). While we did not account for these effects, Moss et al., (2021) showed that the morphology of the T wave remains similar under control conditions. Thus, for the purpose of this study, we do not expect major differences compared to incorporating contraction effects. Nevertheless, this may not be the case when the contraction is abnormal or irregular. Another possible source of error is the use of truncated biventricular anatomies; nevertheless, the valvular section of the ventricles is unlikely to have a sufficient contribution to the 12-lead ECG to change its morphology given the standard orientation of the electrodes. Future studies could consider using closed biventricular geometries and either electromechanical simulations or prescribe the cardiac deformation as extracted from cine MRI data to improve the relevance of matching to the clinical ECGs.

### 4.3 Open-source contributions to the research community

In this paper, we provide an open-source cardiac digital twin pipeline (details of the pipeline can be found in online supplement E.1, flowchart at Figure E.1). Our toolkit includes implementations of the Eikonal model, the pseudo-diffusion reaction-Eikonal model with the ToR-ORd model embedded, the ECG simulation equations, functions for global sensitivity analysis, and the SMC-ABC inference algorithms. These are freely available on GitHub at https://github.com/juliacamps/Cardiac-Digital-Twin. These functionalities have been coded in a modular fashion for easy of translation to other applications. The personalised digital twins of the three healthy subjects, including the mesh and microstructure, inferred Purkinje tree, conduction velocity, and the G_Ks_ field required to reproduce the APD heterogeneity are available at (HERE WE WILL INCLUDE A LINK TO THESE DATA AFTER THE REVIEW PROCESS)..

## 5 Conclusions

We present a novel open-source pipeline for generating cardiac digital twins for virtual therapy evaluation. This pipeline generates biophysically detailed cardiac digital twins from MRI and ECG data with representations of electrophysiological uncertainty. The digital twins were capable of reproducing subject-specific ECG phenotypes and predicting clinically expected behaviour when simulating the effects of Dofetilide at different doses. Our pipeline automatically propagates uncertainty in the final population to *in silico* drug evaluations to inform any decisions based on the simulated outcomes. The development of translation capabilities between reaction-Eikonal and monodomain simulations decreases computational cost while enabling drug safety and efficacy evaluation capabilities, thus enabling scalable digital twinning technology for future expansion and application to *in silico* clinical trials.

## Supplementary Material

Appendix

## Figures and Tables

**Table 1:** Cohort information. Subjects were given IDs by increasing biventricular volume. Columns correspond to Age (years); Sex: Female (F), Male (M); Weight (kg); Body mass index (BMI); Heart rate (HR); Biventricular volume (cm^3^); and Torso volume (dm^3^).

Subject ID	Age	Sex	Weight (kg)	BMI	HR (bpm)	Biventricular volume (cm^3^)	Torso volume (dm^3^)
Subject 1	56	F	53	20.96	66	76	27
Subject 2	76	F	87	33.56	48	107	54
Subject 3	23	M	73	23.84	74	139	35

**Table 4:** Inferred repolarisation parameter-sets (i.e., ‘physiological envelopes’ or digital twins) (Eqs. 24 and 25) for Subjects 1, 2, and 3 (mean ±corrected standard deviation).

	*APD_min_* (ms)	*APD_max_* (ms)	*g_ab_*	*g_pa_*	*g_tv_*	*g_tm_*
Subject 1	189.4 ± 2.2	330.7 ± 5.3	0.4 ± 0.09	−0.83 ± 0.16	0.66 ± 0.15	0.0 ± 0.0
Subject 2	193.6 ± 6.9	366 ± 1.9	0.87 ± 0.15	−0.59 ± 0.11	0.79 ± 0.19	0.04 ± 0.05
Subject 3	187.9 ± 5.1	317.5 ± 6.7	0.64 ± 0.19	−0.73 ± 0.2	0.79 ± 0.19	0.15 ± 0.05

## Highlights

- Novel pipeline for generating cohorts of cardiac digital twins with personalised activation and repolarisation characteristics from clinical MRI and ECG data.
- Human-based pseudo-diffusion reaction-Eikonal implementation to enable fast digital twinning.
- Effective strategy for translation from pseudo-diffusion reaction-Eikonal to monodomain simulations for drug evaluation.
- Parameter uncertainty propagation for virtual therapy testing.
- Compilation of experimental and clinical evidence on ventricular gradients in APD and ionic conductances.
- The cardiac digital twinning pipeline is open-source and freely available.
