# Supplementary material for: Harnessing 12-lead ECG and MRI data to personalise repolarisation profiles in cardiac digital twin models for enhanced virtual drug testing
Source: Med Image Anal. Author manuscript; Available in PMC 2025 Oct 7. (PMC7618211; doi:10.1016/j.media.2024.103361)
Supplement: Appendix [file EMS209271-supplement-Appendix.pdf]

A. Appendix

A.1 Mesh generation and ECG preprocessing

The three subjects' end-diastolic biventricular 3D geometries were generated from their MRI (Zacur et al., 2017; Banerjee et al., 2021). A UNet segments the MRI slices, which are aligned and corrected, providing smooth transitions. We then connect these slices to create the endocardial and epicardial surfaces (imposing 4 mm as the RV's wall thickness). The resulting biventricular geometry was truncated at the base and meshed at a resolution of ~1.5 mm for pseudo-diffusion reaction-Eikonal simulations, ~0.25 mm for demonstrating the translation to monodomain simulations, and ~0.5 mm for the monodomain virtual drug evaluations.

The electrodes were located using a rule-based strategy that derives the locations from a 3D torso geometry (Zacur et al., 2017). These torso geometries were obtained from the MRI 'localiser' (or 'scout') slices, typically acquired to align the MRI machine with the subject's heart. The 'localiser' slices were segmented and fitted to a torso statistical shape model, which produced the required 3D torsos used to estimate the electrode locations.

We pre-processed the subject's clinical 12-lead ECG recordings (Lyon et al., 2018) by removing high-frequency noise using a low-pass Butterworth filter with a 45 Hz cut-off, the baseline drift using a cubic spline method, and power artefacts using a notch filter at 50 Hz. We then aligned the delineated beats (Martinez et al., 2004) with respect to the QRS complex (Woody, 1967), and averaged them. Then, we manually aligned the averaged recordings across all leads to start at the QRS onset to produce the target 12-lead ECG recordings considered for the inference. These clinical ECGs were normalised with respect to the R wave amplitude as in Camps et al., (2024).

A.2 Pseudo-ECG calculation

The pseudo-ECG method models the heart as if it is immersed in an infinite volume isotropic homogeneously conductive medium to enable computing unipolar extracellular potentials using an integral formulation Eq. (26). Comparisons of this method with finite volume conductor methods (Ogiermann et al., 2021) and with heterogeneous conductivity models (Bishop & Plank, 2011) with various organs show mainly amplitude

differences with only minor differences in QRS morphology (Keller et al., 2010). Body surface potentials Φ, were calculated at the electrode locations $(\boldsymbol{x}')$ using:

$$\Phi(\boldsymbol{x}') = \frac{1}{\mathrm{C_m}\chi} \sum_{j=1}^{\mathrm{N}_{src}} -\boldsymbol{\sigma}_j (\nabla U)_j \left[\nabla \frac{b_j}{r_j} \right], \tag{26}$$

where $(\nabla U)_j$ is the spatial gradient of the membrane potential over the j th tetrahedral element, $\boldsymbol{\sigma}_j$ is the conductivity tensor at the j-th element, b_j is the normalised volume scaling factor for the j-th element, r_j is the Euclidean distance from the centroid of the j-th element to the electrode location$(\boldsymbol{x}')$, and N_{src} is the total number of tetrahedral source elements. We used these unipolar electrograms to calculate the three Einthoven leads (i.e., limb leads) and the Wilson leads (i.e., precordial).

When evaluating the ECG recordings from the transmembrane potential simulations, we consider the conductivity tensor $\boldsymbol{\sigma}_j$ to be the identity matrix since our pseudo-diffusion reaction-Eikonal does not include an explicit conductivity term. We demonstrate that the effect of this simplification is small enough to enable the later translation from our inferred parameters to simulate ECGs using the monodomain diffusive term (online supplemental, Fig. E.2).

A.3 Inference hyperparameters

The SMC-ABC strategy is as proposed in Camps et al., (2021) This algorithm iteratively evolves a distribution of parameter sets (approximate posteriors), like the SMC algorithm, while decreasing its cut-off discrepancy, like the ABC algorithm. In our context, the SMC-ABC is similar to an optimisation algorithm that is called iteratively, each time using the resulting distribution of the previous iteration and adjusting the current iteration's cut-off discrepancy gradually to make each iteration manageable. This algorithm converges to multiple local minima in the parameter space and terminates when the current cut-off discrepancy matches the target one. The SMC-ABC returns a distribution of parameter sets, which represents the problem's uncertainty. We directly use this distribution of parameter-sets as our digital twin (i.e., 'physiological envelope') that can be later used to obtain uncertainty in virtual therapy evaluations.

The most relevant hyperparameters of the inference algorithm for this study were: 1) the population size, which determines the number of parameter-sets used by the algorithm, this hyperparameter affects the coverage of the parameter space; 2) the sampling rate per iteration, which determines what proportion of parameter-sets' distribution gets ‘jiggled’ at each iteration by the Markov Chain Monte Carlo algorithm, which affects the convergence speed of the inference process; 3) the target discrepancy cut-off, which controls how well the inferred parameter-sets in the ‘physiological envelope’ need to match the clinical ECG signals to terminate the process due to succeeding at matching the clinical data; and 4) the uniqueness threshold, which controls the minimum variability in the inferred population before terminating the process due to converging.

A.4 APD and ionic channel heterogeneity

Table A.1: Ventricular spatial heterogeneity in ionic current magnitude and ionic channel subunit expression from experimental recordings in both human and canine. These data were collected using combinations of three techniques: voltage-clamp, western blotting, and reverse transcription polymerase chain reaction (RT-PCR).

Ionic currents	Species	Preparation	Channel expression	Peak current and tail current magnitude
I_{to}	Human data (n=6, healthy hearts) (Näbauer et al., 1996)	voltage-clamp	Epi > Endo	
	Human expression data (n=7), canine functional recordings (n=7 myocytes) (Szentadrassy et al., 2005)	Western blotting in human, voltage-clamp for canine	Kv1.4: 40% base/apex ratio (apex > base), KChIP2: 75% base/apex ratio (apex > base) Kv4.3: No base/apex difference	Canine: peak current apex > base (29.6 +- 5.7 pA/pF vs 16.5 +- 4.4 pA/pF)

	Human expression data (n=5), canine functional recordings (n=6 myocytes) (Szabó et al., 2005)	Western blotting in human, voltage-clamp for canine	Epi > Mid	
	Human RT-PCR data (n=15) (Gaborit et al., 2007)	RT-PCR data, confirmed by Western blotting	Epi > Endo (KChIP2)	
	Human RT-PCR data (n=7, non-failing hearts) (Soltysinska et al., 2009)	RT-PCR data confirmed by Western blotting	Epi > Mid > Endo (KChIP2)	
	Human (n=1 normal donor heart) (Opthof et al., 2017)	Langendorff perfusion	LV > RV (Kv1.4)<br>Epi > Endo (KChIP2)	-
I_{Ks}	Human expression data (n=7), canine functional recordings (n=7 myocytes) (Szentadrassy et al., 2005)	Western blotting in human, voltage-clamp for canine	KvLQT1: 40% base/apex ratio (apex > base)<br>MinK: 70% base/apex ratio (apex > base)	Canine: Both peak and tail currents apex > base (5.61 vs 2.14 pA/pF, 1.65 vs 0.85 pA/pV)
	Human expression data (n=5), canine functional recordings (n=6	Western blotting in human, voltage-clamp for canine,	Epi > Mid	

	myocytes) (Szabó et al., 2005)	comparing EPI and MID regions		
	Human RT-PCR data (n=7, non-failing hearts) (Soltysinska et al., 2009)	RT-PCR data	Mid > Endo (KCNE1)<br>Mid > Epi (KCNE1)	
	Human (n=1 normal donor heart) (Opthof et al., 2017)	Langendorff perfusion	Septum > RV > LV (KvLQT1)<br>Septum > LV > RV (MinK)	-
I_{Kr}	Human expression data (n=7), canine functional recordings (n=7 myocytes) (Szentadrassy et al., 2005)	Western blotting in human, voltage-clamp for canine	hERG: No base/apex difference,<br>MiRP1: No base/apex difference	Canine: tail current no base to apex difference
	Human expression data (n=5), canine functional recordings (n=6 myocytes) (Szabó et al., 2005)	Western blotting in human, voltage-clamp for canine	There was no significant difference between Epi and Mid	
	Human (n=1 normal donor heart) (Opthof et al., 2017)	Langendorff perfusion	hERG: LV > RV<br>MiRP1: No difference in any direction	-

I_{K1}	Human expression data (n=7), canine functional recordings (n=7 myocytes) (Szentadrassy et al., 2005)	Western blotting in human, voltage-clamp for canine	Kir2.1: No base/apex difference	Canine: peak current above 40 mV membrane potential apex > base
	Human expression data (n=5), canine functional recordings (n=6 myocytes) (Szabó et al., 2005)	Western blotting in human, voltage-clamp for canine	No significant difference between Epi and Mid	
	Human RT-PCR data (n=7, non-failing hearts) (Soltysinska et al., 2009)	RT-PCR data	Kir2.1 (KCNJ2): Mid > Endo<br>Kir2.1 (KCNJ2): Mid > Epi	
I_{CaL}	Human expression data (n=7), canine functional recordings (n=7 myocytes) (Szentadrassy et al., 2005)	Western blotting in human, voltage-clamp for canine	Alpha_1C: No base/apex difference T	Canine: peak current no base to apex difference
	Human RT-PCR data (n=15) (Gaborit et al., 2007)	RT-PCR	Epi > Endo (Cav1.2)	

	Human RT-PCR data (n=7, non-failing hearts) (Soltysinska et al., 2009)	RT-PCR data	Cav1.2: Mid > Endo<br>Cav1.2: Mid > Epi	
I_{NaCa}	Human RT-PCR data (n=7, non-failing hearts) (Soltysinska et al., 2009)	RT-PCR data	Mid > Endo (NCX1)<br>Mid > Epi (NCX1)	
late INa	Human RT-PCR data (n=15) (Gaborit et al., 2007)	RT-PCR	Endo > Epi (Nav1.5)	
	Human RT-PCR data (n=7, non-failing hearts) (Soltysinska et al., 2009)	RT-PCR data, confirmed by western blotting	Nav1.5 (SCN5A): Mid > Epi<br>Nav1.5 (SCN5A): Endo > Epi	
I_{NaK}	Human RT-PCR data (n=7, non-failing hearts) (Soltysinska et al., 2009)	RT-PCR data	no statistical difference in any direction	
J_{rel}	Human RT-PCR data (n=7, non-failing hearts) (Soltysinska et al., 2009)	RT-PCR data	Mid > Endo (RyR2)<br>Mid > Epi (RyR2)	
J_{up}	Human RT-PCR data (n=15) (Gaborit et al., 2007)	RT-PCR data	Epi > Endo (SERCA2)	
	Human RT-PCR data (n=7, non-failing	RT-PCR data	no statistical difference in any direction	

	hearts) (Soltysinska et al., 2009)			
CMDN	Human RT-PCR data (n=15) (Gaborit et al., 2007)	RT-PCR	Epi > Endo (CALM3)	
I_{KATP}	Human RT-PCR data (n=7, non-failing hearts) (Soltysinska et al., 2009)	RT-PCR data	Mid > Endo (Kir6.2)<br>Mid > Endo (SUR1)<br>Mid > Epi (SUR1)	

Table A.2: Spatial variations in action potential duration (APD), activation recovery intervals (ARIs), and repolarisation time (RT), recorded in the literature in human and canine and showing significant variation in the magnitude and direction of the spatial variation in each of the transmural, apex-to-base, transventricular, and posterior-to-anterior ventricular axes.

Ventricular axes	Species	Preparation	APD or ARI	RT
Transmural	Human (n=10, nine male, one female) (Franz et al., 1987)	In vivo monophasic action potential	-	endo > epi
	Human (n=1 normal donor heart) (Opthof et al., 2017)	Langendorff perfusion, monophasic action potential measurements	endo > mid > epi (275 vs 265 vs 255) (LV, normal T wave)<br>endo > epi (315 vs 188) (septal base LV)<br>epi > endo (315 vs 188) (septal mid)	No differences.

			endo > epi (315 vs 188) (septal apex) endo > epi (315 vs 188) (freewall, mid, and apex)	
	Human (n=1) Brugada syndrome (Coronel et al., 2005)		epi > endo > subepi (280 vs 260 vs 240 ms)	endo > epi (220 ms vs 205 ms)
	Human (n=1) (Conrath et al., 2004)		Endo > Epi (247 ms vs 242 ms)	Epi > Endo (265 ms vs 247 ms)
	Human (n=21) (Taggart et al., 2001)	In vivo transmural plunge electrode	Endo > Mid > Epi (freewall LV) (No significant differences)	
Apex-to-base	Canine (n=7 myocytes) (Szentadrassy et al., 2005)	Ex vivo voltage-clamp measurements	Shorter at the apex by ~50 ms, Base > Apex. Plateau amplitude was lower at the apex by ~15 mV.	-
	Human (n=10) (Chauhan et al., 2006)	In vivo transvenous electrode catheters.	Base > apex > mid (280 vs 270 vs 260 ms) on endo Apex > base (300 vs 270 ms) on epi	Base > apex (320 ms vs 310 ms) on endo, Apex > base (350 vs 340 ms) on epi
	Human (n=10) (Cowan et al., 1988)	In vivo monophasic action potentials	-	Postero-basal was the earliest to repolarise. Implying Apex > Base

	Human (n=1) (Opthof et al., 2017)	Langendorff perfusion	Mid > Base = Apex (252 = 252 vs 188) (septal LV)<br>Base > Mid > Apex (315 vs 252 vs 188) (freewall LV)	Trend (not statistically significant): Apex = Mid > Base (LV and RV, normal T wave)
	Human (n=7, four males, three females) (Ramanathan et al., 2006)	In vivo non-invasive ECGI	Base > Apex (anterior, 240 ms vs 230 ms)<br>Base > Apex (posterior, 290 ms vs 270 ms)	Base > apex (anterior, 280 vs 260 ms)<br>Base > apex (posterior, 260 vs 330 ms)
Transventricular	Human (n=10, nine male, one female) (Franz et al., 1987)	In vivo monophasic action potential	Apico-septal > postero-lateral on endocardium, implying Septum > LV, suggesting a gradient in the direction RV > LV	Trend (no statistical significance): Apico-septal and diaphragmatic (RV lateral) are larger than the rest.
	Human (n=1 normal donor heart) (Opthof et al., 2017)	Langendorff perfusion	LV basal-anterior > Central-posterior RV<br>Implying LV > RV (298 at basal-anterior LV, 200 ms at central-posterior RV).	No differences.
	Human (n=10 with upright T waves) (Cowan et al., 1988)	In vivo monophasic action potentials	-	Anterior and posterior septum is latest to repolarise. Implying Septum > LV/RV
	Human (n=7, four males, three	In vivo non-invasive ECGI	LV > RV (280 vs 250 ms)	LV > RV (340 vs 270 ms)

	females) (Ramanathan et al., 2006)			
	Human (n=15, 11 males, four females) (Bueno-Orovio et al., 2012)	In vivo invasive electrograms using two catheters	LV > RV (208 vs 197 ms)	
Posterior-to-anterior	Human (n=1 normal donor heart) (Opthof et al., 2017)	Langendorff perfusion	Anterior > Posterior (315 vs 188) (basal, LV)	Anterior > Posterior = Lateral (LV only) (315 vs 188 vs 188)
	Human (n=7, four males, three females) (Ramanathan et al., 2006)	In vivo non-invasive ECGI	Posterior > anterior (280 vs 240 ms)	Posterior > anterior (350 vs 270 ms)
	Human (n=10 with upright T waves) (Cowan et al., 1988)	In vivo monophasic action potentials	-	Postero-basal was earliest to repolarise. Implying anterior > posterior

A.5 Computational costs

We computed the average computation time cost of running our pseudo-diffusion reaction-Eikonal and pseudo-ECG for comparison with previous models (Table A.3). We employed the geometry from Subject 3, as this was the one that resulted in higher computation costs from the ones in our three subject cohort. Our implementation of the pseudo-diffusion reaction-Eikonal and pseudo-ECG runs one instance per thread in the CPU of the computer, thus, we ran 128 instances and reported the average time cost for one of them. This was done because our

reaction-Eikonal is implemented to only occupy one thread per parameter-set to enable parallelisation of populations of parameter-sets in any computer with multiple cores.

From these comparison we conclude that our pseudo-diffusion reaction-Eikonal model has a similar computation cost to the no-diffusion-reaction-Eikonal model (Neic et al., 2017), given that the comparison was done in different machines, and while at a lower resolution, our cost remained lower even after also including the cost of calculating the simulated ECG.

Table A.3. Comparison of computation times from different electrophysiology propagation models in the literature using non-high-performance-computing resources on biventricular meshes at different discretisation (edge length) resolutions. The pseudo-diffusion reaction-Eikonal's computation time (last row) was computed by averaging the cost of 128 simulations.

Model	Anatomy setup	Machine specifications	Simulation cost for 450 ms
Monodomain (GPU-accelerated) (Sachetto Oliveira et al., 2018) with ECG calculation	Biventricular mesh at 0.5 mm discretization (Subject 3)	Computer with 8 cores and 1 GPU	19 min (1140 seconds)
	Biventricular mesh at 0.25 mm discretization (Subject 3)	Computer with 8 cores and 1 GPU	4 hours (14400 seconds)
Reaction-diffusion-Eikonal (Neic et al., 2017) without ECG calculation	Biventricular mesh at 1 mm discretization	Computer with 16 cores	197 seconds (Gillette et al., 2021)

No-diffusion-reaction-Eikonal (Neic et al., 2017) without ECG	Biventricular mesh at 1 mm discretization	Computer with 16 cores	8 seconds (Gillette et al., 2021)
Pseudo-diffusion reaction-Eikonal with ECG calculation	Biventricular mesh at 1.5 mm discretization (Subject 3)	Computer with 32 cores	6 seconds

The inference was run on a machine with 2 CPUs, each with an AMD EPYC 7313 16-core processor.

Table A.4. Computation costs for the inference of repolarisation properties for all three subjects.

Subject ID	Population size	Sampling rate	Computation cost	Reached cut-off discrepancy / target value	Number of SMC-ABC iterations
Subject 1	120	50%	16.4 hours	3.0 / 0.5	23
Subject 2	120	50%	17.7 hours	0.53 / 0.5	22
Subject 3	120	50%	20 hours	1.04 / 0.5	16

A.6 Calibration of conductivities using TuneCV in MonoAlg3D

Here we report the monodomain calibrated conductivity values to the inferred and prescribed conduction speeds in the pseudo-diffusion reaction-Eikonal simulations. We considered two resolutions for the monodomain simulations. We demonstrated the translation between the pseudo-diffusion reaction-Eikonal simulations to monodomain using a resolution of 0.25 mm (Table A.5), while we considered a resolution of 0.5 mm (Table A.6) to conduct the virtual drug testing experiments with the inferred physiological envelope for each subject. In all

monodomain simulations, the Purkinje tree was simulated at the prescribed to the conduction speed of 300 cm/s in all reaction-Eikonal simulations, and using a resolution of 0.1 mm, in all monodomain simulations. Thus, the Purkinje tissue conductivity had a calibrated value of 0.006 mS/um.

Table A.5. Calibrated values for the conductivities $\boldsymbol{\sigma}$ (Eq. 2) from the inferred conduction speeds using TuneCV, in MonoAlg3D, with a space discretisation of 0.25 mm, which was used for demonstrating the translation from pseudo-diffusion reaction-Eikonal digital twins to monodomain ones (Sections 2.6 and 3.2).

	Purkinje		Fast endocardial				Fibre		Sheet		Normal	
	Speed	Conductivity	Sparse cm/s	Conductivity	Dense cm/s	Conductivity	Speed	Conductivity	Speed	Conductivity	Speed	Conductivity
Subject 1	300 cm/s	0.006 mS/um	133 cm/s	0.000812908 mS/um	168 cm/s	0.00127666 mS/um	65 cm/s	0.000220785 mS/um	36 cm/s	0.0000831569 mS/um	48 cm/s	0.000131592 mS/um
Subject 2	300 cm/s	0.006 mS/um	97 cm/s	0.000450616 mS/um	130 cm/s	0.000778553 mS/um	65 cm/s	0.000220785 mS/um	27 cm/s	0.000054481 mS/um	48 cm/s	0.000131592 mS/um
Subject 3	300 cm/s	0.006 mS/um	105 cm/s	0.000522967 mS/um	153 cm/s	0.001061 mS/um	65 cm/s	0.000220785 mS/um	40 cm/s	0.0000979606 mS/um	48 cm/s	0.000131592 mS/um

Table A.6. Calibrated values for the conductivities $\boldsymbol{\sigma}$ (Eq. 2) from the inferred conduction speeds using TuneCV, in MonoAlg3D, with a space discretisation of 0.5 mm, which was used for the virtual drug evaluations (Sections 2.7 and 3.3).

	Purkinje		Fast endocardial				Fibre		Sheet		Normal	
	Speed	Conductivity	Sparse cm/s	Conductivity	Dense cm/s	Conductivity	Speed	Conductivity	Speed	Conductivity	Speed	Conductivity
Subject 1	300 cm/s	0.006 mS/um	133 cm/s	0.000916514 mS/um	168 cm/s	0.00138889 mS/um	65 cm/s	0.000283505 mS/um	36 cm/s	0.000128656 mS/um	48 cm/s	0.000183456 mS/um
Subject 2	300 cm/s	0.006 mS/um	97 cm/s	0.000534746 mS/um	130 cm/s	0.000880719 mS/um	65 cm/s	0.000283505 mS/um	27 cm/s	0.0000916707 mS/um	48 cm/s	0.000183456 mS/um
Subject 3	300 cm/s	0.006 mS/um	105 cm/s	0.000610572 mS/um	153 cm/s	0.00117342 mS/um	65 cm/s	0.000283505 mS/um	40 cm/s	0.000143111 mS/um	48 cm/s	0.000183456 mS/um

A.7 Additional inference results

Additional results for the inference of repolarisation properties for subjects 1 and 3 are hereafter presented.

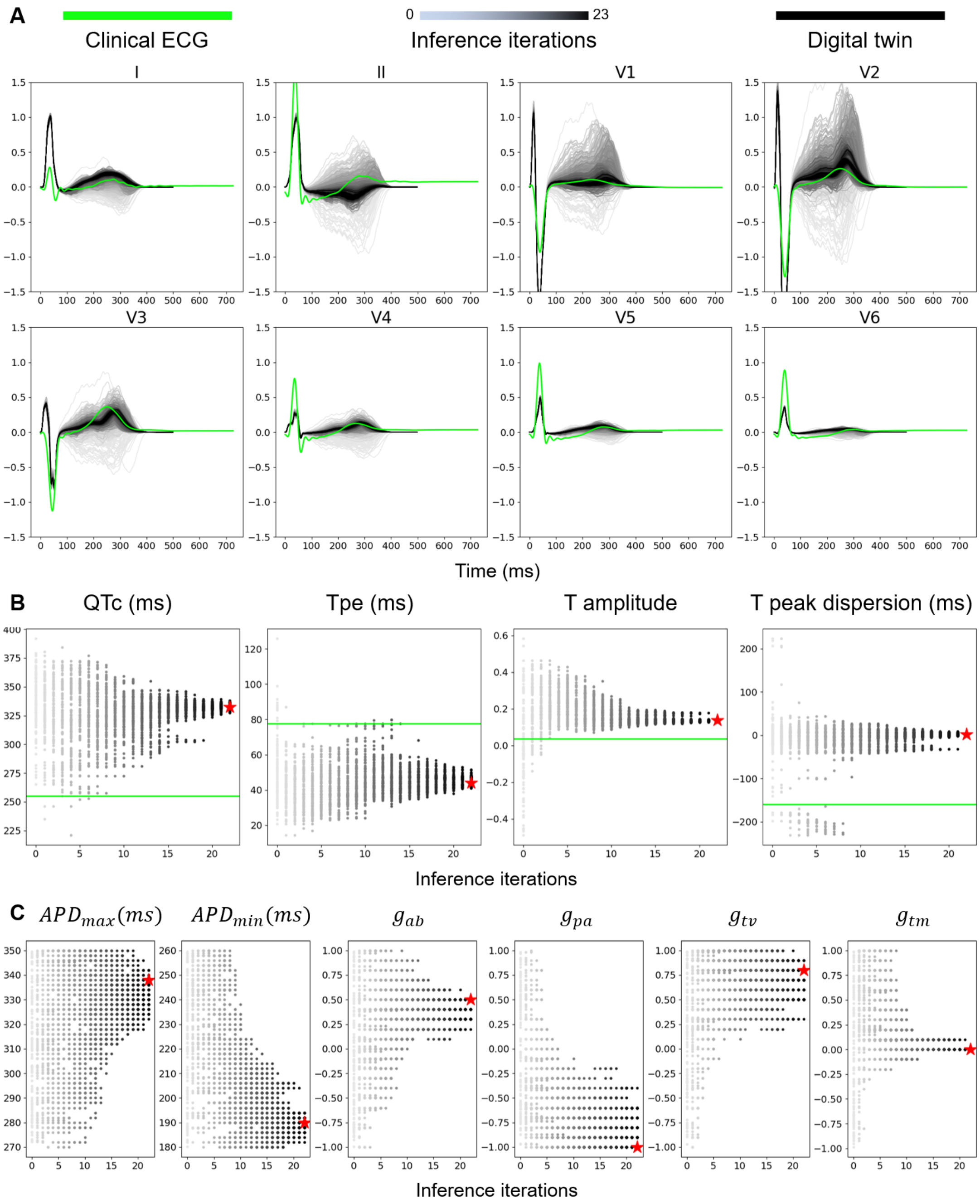

Figure A.1. Inference (Subject 1) iterations effectively explore T wave biomarker space. Clinical subject ECGs are shown in green, successive inference iterations are shown in increasing blackness, and the 'digital twin' (final inferred population) is shown in black. A) The range of T wave morphologies explored by the inference process, converging on the population with the best match to clinical data. B) QT interval, T peak to T end interval (Tpe), average T wave amplitude, and dispersion of T peak timing between leads V3 and V5 converging over successive inference iterations (grey to black) to match clinical values (marked in green horizontal line). C) Progression of the parameter space over successive inference iterations. This parameter space was composed of $APD_{\max}$ (maximum action potential duration), APD_{min} (minimum action potential duration), g_{ab} (APD gradient in the apex-to-base direction), g_{pa} (APD gradient in the posterior-to-anterior direction), g_{tv} (APD gradient in the transventricular direction) and g_{tm} (APD gradient in the transmural direction). The cyan stars in panels B and C indicate the parameter-set with the lowest discrepancy. Inference hyperparameter values: population size = 120, sampling rate = 50%, and target discrepancy cut-off = 0.5, uniqueness termination threshold = 50%.

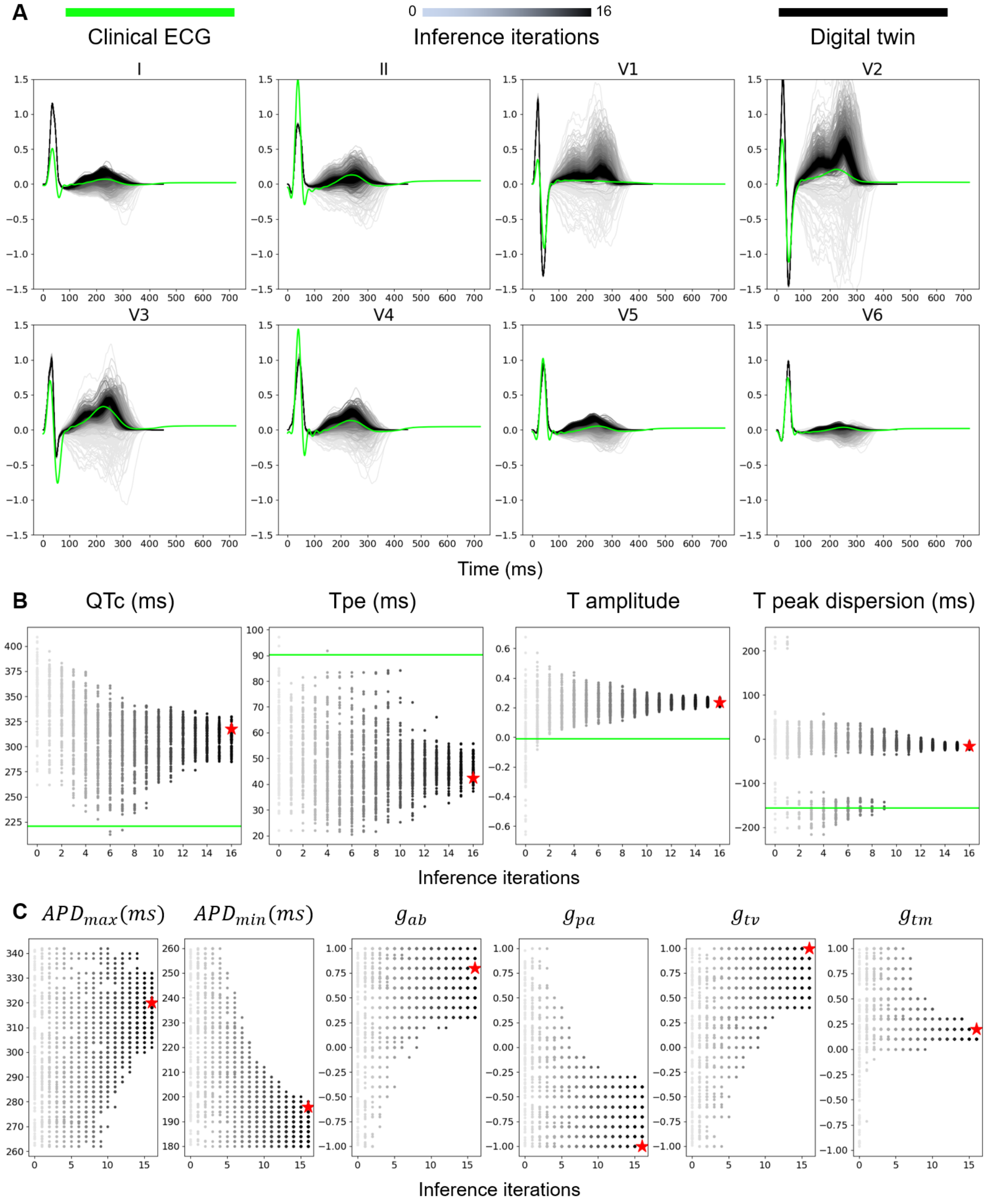

Figure A.2. Inference (Subject 3) iterations effectively explore T wave biomarker space. Clinical subject ECGs are shown in green, successive inference iterations are shown in increasing blackness, and the 'digital twin' (final inferred population) is shown in black. A) The range of T wave morphologies explored by the inference process,

converging on the population with the best match to clinical data. B) QT interval, T peak to T end interval (Tpe), average T wave amplitude, and dispersion of T peak timing between leads V3 and V5 converging over successive inference iterations (grey to black) to match clinical values (marked in green horizontal line). C) Progression of the parameter space over successive inference iterations. This parameter space was composed of APD_{max} (maximum action potential duration), APD_{min} (minimum action potential duration), g_{ab} (APD gradient in the apex-to-base direction), g_{pa} (APD gradient in the posterior-to-anterior direction), g_{tv} (APD gradient in the transventricular direction) and g_{tm} (APD gradient in the transmural direction). The cyan stars in panels B and C indicate the parameter-set with the lowest discrepancy. Inference hyperparameter values: population size = 120, sampling rate = 50%, and target discrepancy cut-off = 0.5, uniqueness termination threshold = 50%.

A.8 Global sensitivity analysis results

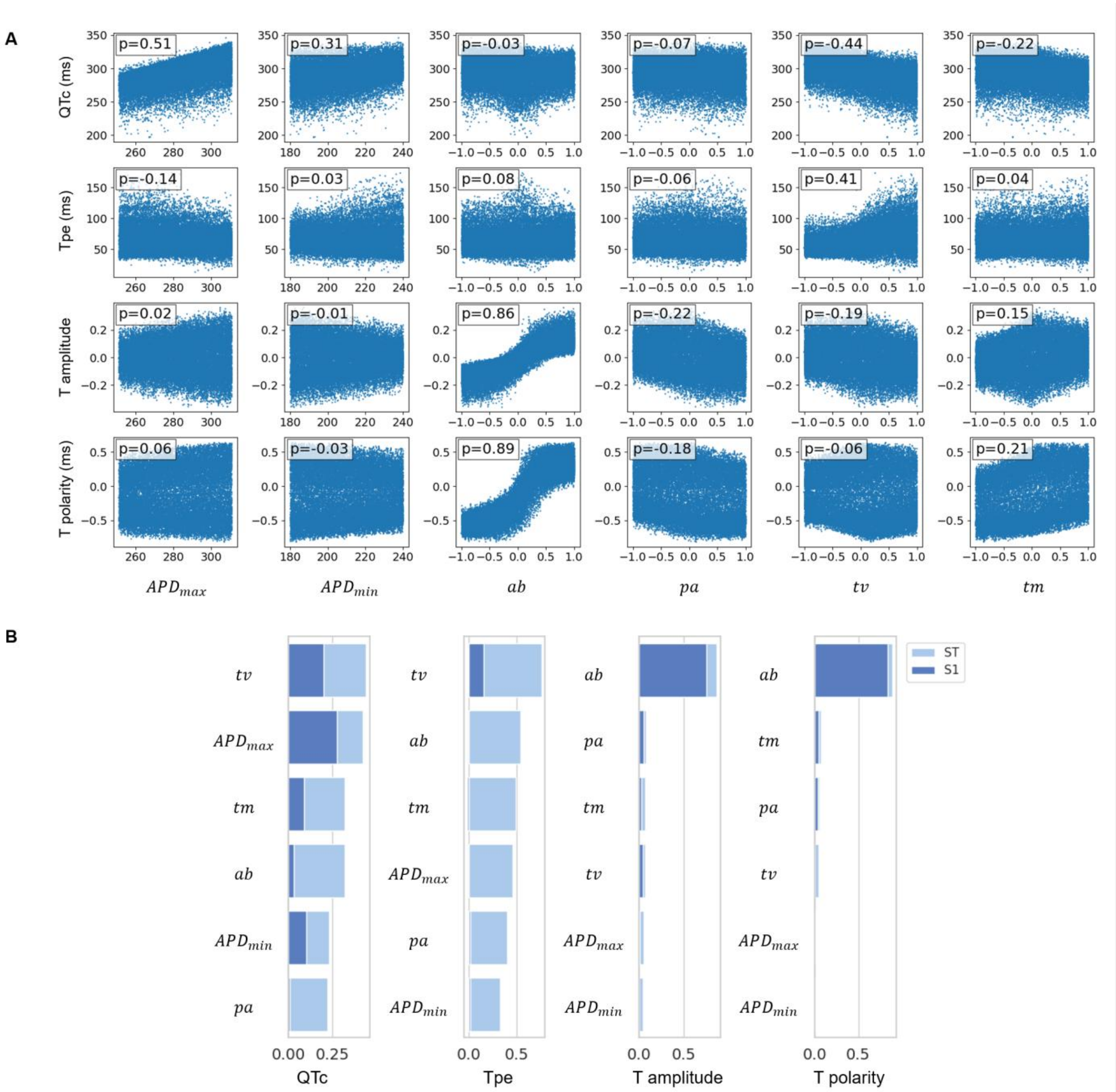

Figure A.3. Sensitivity of T wave biomarkers to APD spatial heterogeneities. (A) Scatter plot (n=28672) with Pearson's correlations of QT interval, T peak to T end (Tpe) interval, T wave amplitude, T wave polarity, and dispersion of T peak timing between leads V3 and V5 to maximum APD (APD_{max}), minimum APD (APD_{min}), apex-

to-base gradient (ab), posterior-to-anterior gradient (pa), transventricular gradient (tv), and transmural gradient parameters (tm). (B) Ranked total Sobol index (ST) and first-order effects index (S1) from global sensitivity analysis.

A.9 Investigations of bifid T waves in reaction-Eikonal simulations

We analysed the effect of the choice of cellular model on the T wave morphology using a unipolar electrogram approximation, as described by Potse et al., (2009). This consisted of subtracting a simulated action potential by itself time-shifted by 20 ms. Gillette et al., (2021) did not report bifid T waves in their simulations. They used the Mitchell-Schaeffer cellular model (MS) (Mitchell & Schaeffer, 2003), which is a two-current action potential shape model. On the other hand, we considered the ToR-ORd (Tomek et al., 2019) cellular model since it has been validated in the context of drug simulations (Tomek et al., 2019). Therefore, we compared the effect of using the ToR-ORd versus the Mitchell-Schaeffer cellular model on T wave morphology (Fig. A.4).

We calculated the first derivative of the simulated action potentials and saw non-monotonic changes in the gradient of the ToR-ORd model that are significantly less pronounced in the MS model (Fig. A.4, first column). We then approximated unipolar electrograms for the ToR-ORd and MS cell models by calculating the difference between a baseline action potential and a time-shifted action potential that has been delayed by 20 ms (Fig. A.4 second column). The ToR-ORd unipolar electrogram showed the bifid morphology while the MS one did not, highlighting the effect of the non-monotonic gradient of the ToR-ORd action potential on the ECG. We also repeated the inference process using either the MS or ToR-ORd models and showed that the simulated ECGs were only notched when using ToR-ORd (Fig. A.4 third column).

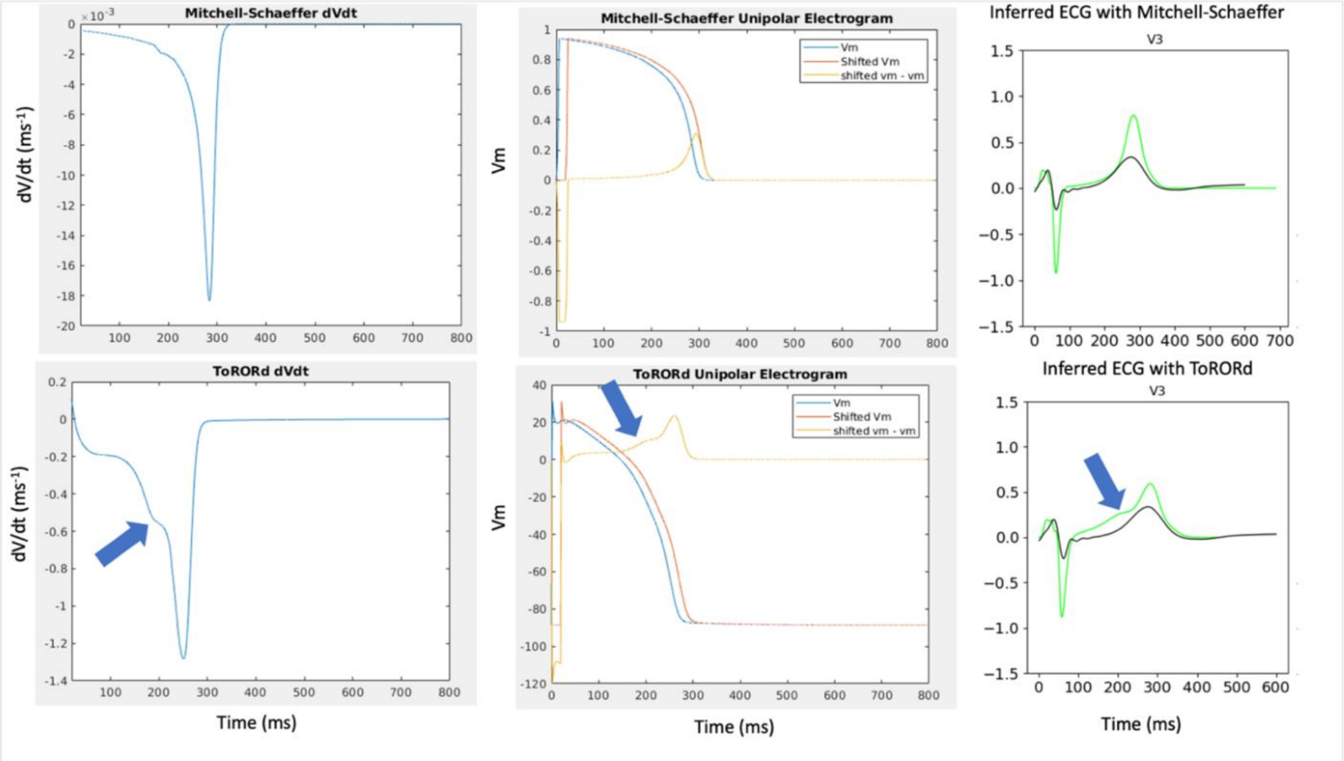

Figure A.4: Evaluation of the first derivative over time of the ToR-ORd (first column – bottom row) and Mitchell-Schaeffer (MS) (first column – top row) action potentials and saw non-monotonic changes in the gradient of the ToR-ORd model that is significantly less pronounced in the MS model. Furthermore, we approximated unipolar electrograms for the ToR-ORd (second column – bottom row) and MS (second column – top row) cell models by calculating the difference between a baseline action potential and a time-shifted action potential that has been delayed by 20 ms. The simulated ToR-ORd unipolar electrogram showed the bifid morphology while the MS one (green – third column, top row) did not, highlighting the effect of the non-monotonic gradient of the ToR-ORd action potential on the ECG. We show simulated ECG signals (lead V3), using the pseudo-diffusion reaction-Eikonal model (green – third column) for Subject 2 from the inference results compared to the clinical signal (black – third column) for comparison. The top row of the last column shows the simulated ECG (green) using the MS model, while the bottom row shows the simulated ECG with the ToR-ORd (green). We use an arrow to point to the appearance of the bifid morphologies in the bottom panels of the figure.
